# Metabolomics’ Change Under β-Cypermethrin Stress and Detoxification Role of *CYP5011A1* in *Tetrahymena thermophila*

**DOI:** 10.3390/metabo15030143

**Published:** 2025-02-20

**Authors:** Wenyong Zhang, Wenliang Lei, Tao Bo, Jing Xu, Wei Wang

**Affiliations:** 1 School of Life Science, Shanxi University, Taiyuan 030006, China; zhangwy@tit.edu.cn (W.Z.); xujing@sxu.edu.cn (J.X.); 2 Key Laboratory of Chemical Biology and Molecular Engineering of Ministry of Education, Institute of Biotechnology, Shanxi University, Taiyuan 030006, China; 202113002002@email.sxu.edu.cn (W.L.); botao@sxu.edu.cn (T.B.); 3 Taiyuan Institute of Technology, Taiyuan 030008, China; 4 Shanxi Key Laboratory of Biotechnology, Taiyuan 030006, China

**Keywords:** β-cypermethrin, *T. thermophila*, metabolomics, *CYP5011A1*

## Abstract

Background: β-cypermethrin (β-CYP) exhibits high toxicity to aquatic organisms and poses significant risks to aquatic ecosystems. *Tetrahymena thermophila*, a protozoa widely distributed in aquatic environments, can tolerate high concentrations of β-cypermethrin. However, the comprehensive detoxification mechanisms remain poorly understood in *Tetrahymena*. Methods: Untargeted metabolomics was used to explore the detoxification mechanisms of *T. thermophila* under β-CYP stress. Results: Trehalose, maltose, glycerol, and D-myo-inositol were upregulated under β-CYP exposure in *Tetrahymena*. Furthermore, the expression level of *CYP5011A1* was upregulated under β-CYP treatment. *CYP5011A1* knockout mutants resulted in a decreasing proliferation rate of *T. thermophila* under β-CYP stress. The valine–leucine and isoleucine biosynthesis and glycine–serine and threonine metabolism were significantly affected, with significantly changed amino acids including serine, isoleucine, and valine. Conclusions: These findings confirmed that *T. thermophila* develops β-CYP tolerance by carbohydrate metabolism reprogramming and Cyp5011A1 improves cellular adaptations by influencing amino acid metabolisms. Understanding these mechanisms can inform practices aimed at reducing the adverse effects of agricultural chemicals on microbial and environmental health.

## 1. Introduction

Synthetic pyrethroid insecticides have become the predominant choice for managing agricultural pests and controlling public health issues globally over the past decades due to their high toxicity and effectiveness against a range of target organisms and their low toxicity to mammals [1,2,3]. A variety of pyrethroid pesticides have been developed, among which cypermethrin is one of the most commonly used. In the fiscal year 2021–2022, India alone consumed approximately 340.97 metric tons of cypermethrin [1]. Cypermethrin constitutes more than 50% of the pyrethroid market share [4] and is frequently detected in environmental residues in China [5]. Despite the utility of cypermethrin, it exhibits non-selective toxicity and poses risks to non-target organisms through mechanisms such as oxidative stress, apoptosis [6], and inhibition of voltage-gated ion channels [7], among others. The repetitive and uncontrolled application inevitably leads to the contamination of aquatic ecosystems via surface runoff [8], pesticide spraying, and discharges from pesticide factory effluents [9]. This contamination disrupts aquatic ecosystems and adversely affects the growth and reproduction of aquatic species [10,11]. Thus, the accumulation of cypermethrin in aquatic environments has made its use increasingly controversial.

Excessive insecticide use has led to the development of resistance in different organisms by inducing changes in cytochrome P450s expression through various receptor proteins [12]. P450s are known for their involvement in the metabolism of various compounds, including pesticides. Increased expression of certain P450s enhances insect resistance to insecticides by facilitating the detoxification and metabolism of these toxic compounds. For instance, α-cypermethrin (α-CYP) upregulates the expression of *CYP9A105* in the midguts of *Spodoptera exigua* in a dose-dependent manner, while silencing *CYP9A105* increases mortality [13]. Knockdown of *CYP6A2* significantly enhances the sensitivity of *Aphis gossypii* to α-CYP [14]. Overexpression of *CYP6P5* confers resistance to α-CYP in *Drosophila melanogaster*, whereas the inhibition of P450 by piperonylbutoxide significantly increases the mortality rate of *Drosophila melanogaster* [15]. In *Spodoptera litura*, the 24 h mortality rate dramatically increases when treated with an LC_30_ concentration of β-cypermethrin (β-CYP), and the expression levels of *CYP6AE43* and *CYP6AE48* are reduced, either simultaneously or individually [16]. Cytochrome P450 inhibitors potentiate the cytotoxicity of cypermethrin to hepatocytes, while co-incubation with P450 inducers reduces hepatocytes susceptibility to cypermethrin, highlighting the role of P450s in cypermethrin detoxification [17].

The β-CYP treatment triggered oxidative stress responses in *Tetrahymena thermophila* and led to a decreased proliferation rate with increasing β-CYP concentration [18]. However, the mechanisms bringing about the tolerance to high concentrations of β-CYP in *Tetrahymena* remain underexplored. In *T. thermophila*, 44 putative P450 genes have been identified through whole-genome microarray analyses and classified into 13 families [19]. A recent study showed that Cyp5013C2 efficiently degrades DDT to DDD, resulting in robust tolerance of *T. thermophila* to DDT [20]. Additionally, differentially expressed genes related to drug metabolism, specifically cytochrome P450, under dihydroartemisinin stress suggest that these enzymes play a defensive role against dihydroartemisinin in *T. thermophila* [21]. Transcriptomic analysis of *T. thermophila* exposed to β-CYP stress showed an upregulation of *CYP5011A1* (TTHERM_00527100), a cytochrome P450 monooxygenase (P450) [18], strongly implying that *CYP5011A1* may play an important role in the metabolic detoxification of β-CYP.

In this study, metabolic perturbations in *T. thermophila* were analyzed using a non-targeted metabolomics approach. *CYP5011A1* knockout mutants were then constructed. Metabolic profiling in the *CYP5011A1* knockout mutants was further analyzed to explore the metabolic basis of β-CYP tolerance in *T. thermophila* and the role of *CYP5011A1* in this tolerance. The study provides valuable insights for developing strategies to mitigate the impact of pesticides on non-target organisms and ecosystems.

## 2. Materials and Methods

### 2.1. Chemicals and Reagents

β-CYP (purity > 98%) from Guangdong Liwei Chemical Industry Co., Ltd. (Maoming, China) was dissolved in dimethyl sulfoxide (DMSO) to prepare a mother solution with a concentration of 50 g/L. Chromatographically pure methanol, ethanol, deuterium-labeled succinic acid, methoxyamine hydrochloride, N-methyl-n -(trimethylsilyl) trifluoroacetamide (MSTFA) with 1% trimethylchlorosilane (TMCS), and trimethoxy silane were purchased from Merck (Darmstadt, Germany). TRIeasy^TM^ Total RNA Extraction Reagent and Hieff^®^ qPCR SYBR Green Master Mix (High Rox Plus) were obtained from Yeasen Biotechnology Co., Ltd. (Shanghai, China). The Goldenstar^TM^ RT6 cDNA Synthesis kit was purchased from Tsingke Biotechnology Co., Ltd. (Beijing, China). Penicillin G, streptomycin sulfate and amphotericin B were obtained from Beijing Solarbio Science & Technology Co., Ltd. (Beijing, China). Restriction endonucleases were from Thermo Fisher Scientific Inc. (Waltham, MA, USA). Other analytical grade reagents were purchased from Sangon Biotech (Shanghai, China).

### 2.2. Culture of T. thermophila

The *T. thermophila* B2086 were acquired from the National Tetrahymena Stock Center (http://tetrahymena.vet.cornell.edu/, accessed on 12 September 2017) and cultured in super proteose peptone (SPP) medium (1% proteose peptone, 0.2% D-Glucose, 0.1% yeast extract, 0.003% Fe-EDTA, 100 units/mL penicillin G, 100 mg/L streptomycin sulfate, 0.025 mg/L amphotericin B, and pH 7.4) at 30 °C with shaking at 135 rpm. For β-CYP exposure analysis, cells were inoculated into SPP containing β-CYP, starting with an initial density of 2 × 10^4^ cells/mL, and cells exposed to an equal volume of DMSO served as the control group.

### 2.3. Metabolic Perturbations Analysis in T. thermophila

B2086 was cultured in a new sterile SPP medium containing either 25 mg/L (C25) or 125 mg/L (C125) β-CYP for 24 h, and cells exposed to DMSO served as the control group (CK). An amount of 50 mL of cell culture solution was centrifugated at 3000 rpm for 5 min to collect the cells. The cell pellet was washed three times with 10 mmol/L Tris-HCl buffer (pH 7.4) and subsequently quenched with liquid nitrogen immediately.

Metabolite extraction was performed as previously described [22]. Briefly, cells were ground thoroughly in liquid nitrogen for 10 min. An amount of 50 mg of the ground cell powder was suspended in 1 mL 50% methanol extraction buffer. The suspension was then centrifuged at 10,000 rpm for 5 min at 4 °C to obtain supernatant. Subsequently, 200 μL supernatant was mixed with 3 μL internal standard solution (succinic-*d4* acid, 1.4 mg/mL). The mixture was vacuum-dried (Heto PowerDry PL3000, Thermo Electron Corporation, Waltham, MA, USA) and stored at −80 °C for further derivatization. Two-stage derivatized procedure was conducted [22]. The resulting supernatant (100 μL) was transferred into a vial and stored at −40 °C after centrifugation at 10,000 rpm for 5 min. For each treatment group, biological replicates were shown independently (*n* = 6). Equal volumes of samples to be measured were pooled to prepare quality control samples (QC), which were evenly inserted into the analysis sample sequence and analyzed together with the samples on the GC-MS to evaluate the stability during analysis.

Metabolite profiling in *T. thermophila* was conducted using an Agilent 5975C MSD mass spectrometer coupled with an Agilent 7890A Gas Chromatograph equipped with a Triple-Axis Detector (Agilent Technologies, Santa Clara, CA, USA) and an Agilent J&W HP-5 capillary column (30 m × 0.25 mm i.d., 0.25 μm film thickness). High-purity helium served as the carrier gas, set at a flow rate of 1 mL/min. Each sample (1 μL) was injected with a split ratio of 1:10 and the injection temperature was maintained at 280 °C. The gas chromatography oven temperature was kept at 70 °C for 2 min, then raised to 290 °C at a rate of 5 °C/min and maintained for 3 min. The running time was set to 49 min. The mass spectrometer was operated in electron impact ionization (70 eV) mode with full scan mode (50–800 *m*/*z*). The ion source and interface temperature were set to 250 °C and 280 °C, respectively. Data acquisition was performed using MSD Productivity ChemStation (version E.0201.1177, Agilent Technologies).

The raw MS data were first processed through baseline filtering, peak identification, peak matching, retention time correction, alignment, deconvolution, and peak feature extraction. Then, the metabolites’ data matrix was identified by comparing it with the National Institute of Standards and Technology (NIST11.0) standard library for the qualitative and quantitative analysis of metabolites. Features with >50% missing values in the metabolites’ data matrix were removed and the remaining missing values were estimated with mean values. According to the response intensity of the added internal standard in different retention times, the metabolites’ peak areas were normalized against the internal standard peak area. The quantified data matrix included metabolites’ name, class, compound ID from Human Metabolome Database (HMDB), and normalized metabolites’ level data were output into Excel format: Metabolites’ data.xlsx file (Supplementary Materials). The quantified data were subjected to MetaboAnalyst 6.0 (https://www.metaboanalyst.ca/, accessed on 4 June 2024) for pareto scaling and normalization followed by multivariate statistical, including unsupervised principal components analysis (PCA) and orthogonal partial least-square discriminant analysis (OPLS-DA) between two groups. The response permutation testing was used to evaluate the robustness of the model. On the basis of the variable importance in the projection (VIP) value of each variable (VIP > 1.0) in the OPLS-DA model, |log_2_(FC)| > 1, and *p*-value < 0.05 from two-tailed Student’s T-test between two treatment groups, differential metabolites and potential biomarkers were identified. Fold change and two-tailed Student’s T-test were performed using Microsoft Excel 365 Personal (Redmond, WA, USA). Volcano plot and Veen plot were constructed using Origin 2011, and clustered heatmap diagram was constructed using MetaboAnalyst 6.0.

Pathway and KEGG analysis of the differential metabolites were performed using MetaboAnalyst 6.0 (https://www.metaboanalyst.ca/, accessed on 21 June 2024) based on its affinis *Paramecium tetraurelia* pathway library and Fisher’s Exact Test enrichment method. Pathways with *p* < 0.05 were considered as statistically significant.

### 2.4. Construction of CYP5011A1 Knockout and HA-Tagged Mutants

To investigate the role of *CYP5011A1* in *T. thermophila* under β-CYP exposure, both *CYP5011A1* knockout and HA-tagged mutants were constructed. For the construction of *CYP5011A1* knockout mutants, 675 bp 5′ flanking sequences and 506 bp 3′ flanking sequences were amplified using primers pairs KO-*CYP5011A1*-5′F/R and KO-*CYP5011A1*-3′F/R, respectively (Table S1). Subsequently, the flanking sequences were inserted into the vector pNEO4 using the Hieff Clone Plus One Step Cloning Kit (YEASEN, Shanghai, China) to create targeting recombinant plasmids. Following linearization with restriction enzymes *BamH* I/*Xho* I, the recombinant plasmids containing the neo4 cassette were transferred into starved B2086 cells using a biolistic gun GJ-1000 (SCIENTZ, Ningbo, China) [18]. Selection of *CYP5011A1* knockout mutants was conducted under a paromomycin concentration gradient, and mutants were confirmed by PCR and qRT-PCR using primers JD-KO-*CYP5011A1*F/R and q*CYP5011A1*F/R (Table S1), respectively. An amount of 17S rRNA served as the endogenous control for qRT-PCR, and the total RNA extraction, cDNA synthesis, and qRT-PCR reaction system were performed following established protocols [18].

For HA-tagged mutants, a 1536 bp fragment of *CYP5011A1* was amplified from genomic DNA using primers OE-*CYP5011A1*F/R (Table S1). After sequencing verification, the fragment was inserted downstream of the HA-tag in the p*XS75* vector, which contains the flanking region of the gene *MTT1* (Metallothionein 1) from *T. thermophila*. The recombinant vectors were linearized with restriction enzymes *Xho* I/*Sac* I and transferred into starved B2086 cells using a biolistic gun GJ-1000. After selection under a paromomycin concentration gradient and identification by PCR with primers JD-MTT1F/R (Table S1), the mutants HA-*TtCYP5011A1* were obtained.

### 2.5. Subcellular Localization of HA-Cyp5011A1

Cells induced with 0.1 μg/mL Cd^2+^ for 24 h were collected and fixed with Lavdowsky fixing solution (ethanol:formaldehyde:acetic acid:water = 50:10:1:39) at 4 °C for fixation. Immunofluorescent localization slides were prepared according to established protocols [18] and incubated with anti-HA rabbit mAb (1:300 dilution, #3724S, CST, Danvers, MA, USA) and goat anti-rabbit IgG/FITC secondary antibody (1:600, AQ132F, Millipore, Billerica, MA, USA). The slides were subsequently imaged using a Delta Vision microscope (GE Healthcare, General Electric Company, Boston, MA, USA).

### 2.6. Assay of β-CYP Toxicity to CYP5011A1 Mutants

Mid-logarithmic phase cells (2.5–3 × 10^5^ cells/mL) were inoculated into fresh sterile toxicity medium at a final density of 2.0 × 10^4^ cells/mL. Toxicological analysis was conducted using SPP medium containing 250 mg/L β-CYP. Cell densities were sampled at regular intervals and fixed by 0.75% trichloroacetic acid. Cell number was measured using a hemocytometer and generation time (GT) between binary division (3–18 h) of cells was analyzed [18].

### 2.7. Metabolic Perturbations Analysis in cyp5011A1KO

To analyze the effect of *CYP5011A1* knockout on the tolerance of *T. thermophila* to β-CYP, the *cyp5011A1*KO strains were cultured in a sterile SPP medium containing 125 mg/L β-CYP or DMSO for 24 h. Metabolite extraction and GC-MS analysis were performed as described in Section 2.3. Wild-type (WT) cells exposed to an equal volume of DMSO were used as the control group. The *cyp5011A1*KO strain exposed to DMSO was marked as the KO group, and the cyp5011A1KO strain exposed to 125 mg/L β-CYP was marked as the KO-cyp group. Equal volumes of each sample were pooled to serve as quality control (QC). Biological replicates of each treatment group were analyzed separately (*n* = 6).

### 2.8. Statistics Analysis

Statistical analyses were performed using SPSS 22.0 (International Business Machines Corporation, Armonk, NY, USA). One-way analysis of variance (ANOVA) followed by post hoc multiple comparisons (Tukey or Tamhane) was used to compare data among multiple groups. Significant differences (*p* < 0.05) among different groups were indicated with different letters.

## 3. Results

### 3.1. Metabolic Profile Changed in T. thermophila Exposed to β-CYP

**Figure 1:**
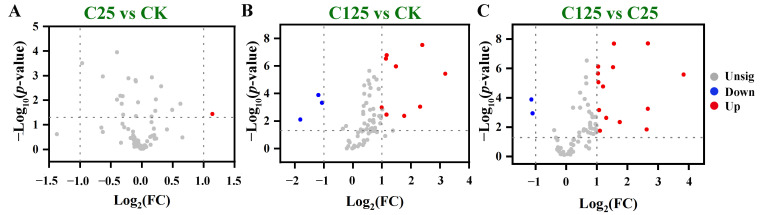
Volcano plots of metabolites’ distribution between two comparison groups. (**A**) C25 vs CK; (**B**) C125 vs CK; and (**C**) C125 vs C25. Grey node (unsig) labels the metabolite level, which was not significantly different between the two comparison groups; red node (up) labels the metabolite level in the former group, which was significantly upregulated compared with the latter; and the opposite is true for blue node (down). CK refers to wild-type cells exposed to DMSO; C25 refers to wild-type cells exposed to 25 mg/L β-CYP; and C125 refers to wild-type cells exposed to 125 mg/L β-CYP.

The metabolites in *T. thermophila* exposed to varying concentrations of β-CYP were analyzed using GC-MS. In total, 72 metabolites, including 20 amino acids, 11 carbohydrates, 6 nucleic acids, 5 fatty acids, 1 ester, 5 carboxylic acids, 3 amines, and 21 other metabolites were identified (Metabolites’ data.xlsx in Supplementary Materials). Differential metabolites were screened between any two groups based on the following criteria: |log_2_(FC)| > 1 and *p*-value < 0.05 from a two-tailed Student’s T-test. Compared with CK, L-asparagine was significantly upregulated in C25 (Figure 1A, Table S2), while D-fructose, adenosine, and glycoside were significantly downregulated, 9 metabolites including 2-deoxy-6-phosphogluconolactone, L-Chiro-Inositol, linolenic acid, myo-inositol, phosphonic acid, glycerol, trehalose, maltose, and N-acetylaspartylglutamic acid were upregulated in C125 (Figure 1B, Table S2). Comparison of C25 and C125 revealed alterations in 16 metabolites, with D-fructose and glycoside significantly downregulated, and 14 metabolites including 2-deoxy-6-phosphogluconolactone, L-Chiro-Inositol, linolenic acid, myristic acid, myo-inositol, trehalose, phosphonic acid, glycerol, 2-monostearin, beta-D-galactopyranoside, propane, maltose, dodecanoic acid, and aminomethane significantly upregulated at a higher concentration of β-CYP (Figure 1C, Table S2).

The unsupervised PCA model was used to reduce the dimensionality of the data matrix, revealing internal similarities and differences among multiple variables through several principal components. In the PCA scores plot (Figure S1A), QC samples were distributed aggregatively and the percentage of peaks with a coefficient of variation ≤20% accounted for 89.8% of the total number of peaks in the QC samples (Figure S1C), indicating acceptable stability and good repeatability of the analytical methods. The first two principal components accounted for a cumulative variance of 86.1%, with PC1 explaining 45.3% and PC2 explaining 40.8% of the variance, respectively. A distinct separation of data points was observed between C125 and CK, as well as between C125 and C25, indicating significant metabolic changes in the *T. thermophila* exposed to higher concentrations of β-CYP.

**Figure 2:**
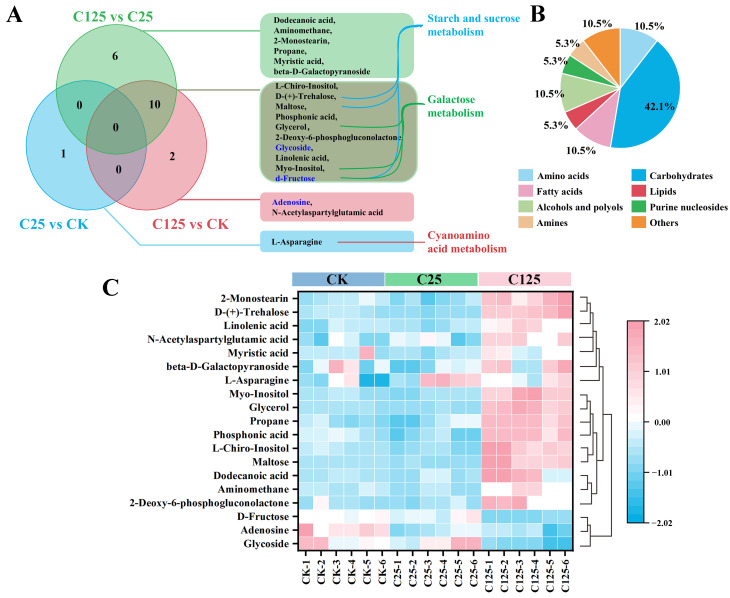
Venn diagram, pie chart of classes, and hierarchically clustered heatmap of significantly changed metabolites (SCMs). (**A**) Venn diagram of differential metabolites. The corresponding differential metabolites are listed in the rounded rectangles. Metabolites in blue text indicate metabolite levels are under-regulated in the former group compared with those in the latter group. Metabolites being linked to metabolic pathway indicate that these metabolites are involve in the metabolic pathway. (**B**) The pie chart of classes of differential metabolites. (**C**) Hierarchically clustered heatmap of SCMs in CK, C25, and C125. Hierarchical clustering of 19 SCMs (*p* < 0.05, VIP > 1, and |log_2_FC| > 1) in *T. thermophila* under exposure to β-CYP using the normalized and pareto-scaled relative metabolite data. The color scale from blue to red indicates relative metabolite levels, from low to high.

The OPLS-DA model is a multivariate statistical analysis method with supervised pattern recognition that maximizes the separation between groups and helps identify variables carrying class-separating information. To effectively eliminate the influence of metabolites unrelated to the study and identify differential metabolites and potential biomarkers, the OPLS-DA model was applied between two different treatment groups. The R^2^Y values for OPLS-DA models of C25 vs. CK, C125 vs. CK, and C125 vs. C25 were 0.917, 0.96, and 0.963, respectively. Q^2^ values for OPLS-DA models of C25 vs. CK, C125 vs. CK, and C125 vs. C25 were 0.733, 0.93, and 0.936. The models demonstrated robust predictive ability, with R^2^Y close to 1 and Q^2^ greater than 0.7 (Figure S2). Additionally, *p*-values for all three models were less than 0.05 in permutation tests with 1000 iterations (Figure S3), indicating a good fit [23]. The VIP value in the OPLS-DA model reflects the contribution of each variable to the overall fit degree and classification ability of the model. Based on a VIP value greater than 1, the differential metabolites identified earlier were further refined and, ultimately, a total of 19 significant differential metabolites were identified across the three comparison groups (Figure 2A, Table S2). The relative concentration of significantly changed metabolites (SCMs) displayed a discrepancy between C125 and CK or C125 and C25 (Figure 2C). Classification of these metabolites revealed that carbohydrates, amino acids, fatty acids, and alcohols/polyols were primarily altered, with carbohydrates representing 42.1% of the total SCMs (Figure 2B). No common differential metabolites were identified across all groups; however, specific metabolites such as adenosine and N-acetylaspartylglutamic acid were uniquely altered in C125 vs CK, and dodecanoic acid, aminomethane, 2-monostearin, propane, myristic acid, and beta-D-galactopyranoside were uniquely altered in C125 vs C25. The shared 10 differential metabolites between C125 vs CK and C125 vs C25 were predominantly carbohydrates and polyols, indicating the significant impact of higher β-CYP concentration on carbohydrates and polyols metabolism in *T. thermophila*.

**Figure 3:**
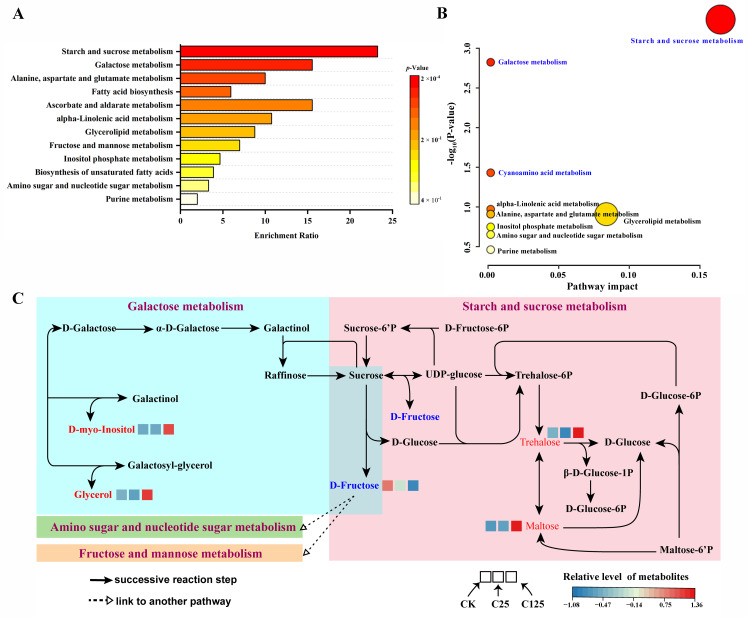
Enrichment analysis and pathway analysis of *T. thermophila* exposed to β-CYP. (**A**) Enrichment analysis. Yellow and red indicate high and low *p*-values, respectively. The horizontal axis represents enrichment ratio. A larger enrichment ratio indicates a greater number of metabolites annotated to the pathway. (**B**) Summary of pathway analysis. Each bubble represents a specific pathway. Bubble size increases with the pathway impact values. Bubble colors represent −log_10_*p*-values, with darker colors indicating more critical metabolic pathways. Pathways with a *p*-value < 0.05 are considered significantly enriched and are labeled with blue text. (**C**) Schematic diagram of key metabolic pathways in *T. thermophila* exposed to different concentrations of β-CYP. Upregulated metabolites are marked in red text and downregulated metabolites in blue text in schematic diagram. Different colored square boxes indicate the main differential metabolite levels in CK, C25, and C125. The black solid arrows represent successive reaction steps in cells, while the dotted hollow arrows represent metabolites linked to other metabolic pathways.

Metabolic pathway and KEGG analysis were conducted to identify significant perturbations in pathways. The results showed that SCMs were significantly enriched in three biological pathways: starch and sucrose metabolism, galactose metabolism, and cyanoamino acid metabolism (Figure 3 and Table 1). Metabolites such as D-fructose, trehalose, and maltose are involved in starch and sucrose metabolism, while D-fructose, glycerol, and myo-inositol are linked to galactose metabolism (Figure 3C). These two pathways belong to carbohydrate metabolism. Additionally, L-asparagine observed in C25 vs CK is involved in cyanoamino acid metabolism.

### 3.2. Knockout of CYP5011A1 Decreased the Tolerance of T. thermophila to β-CYP

**Figure 4:**
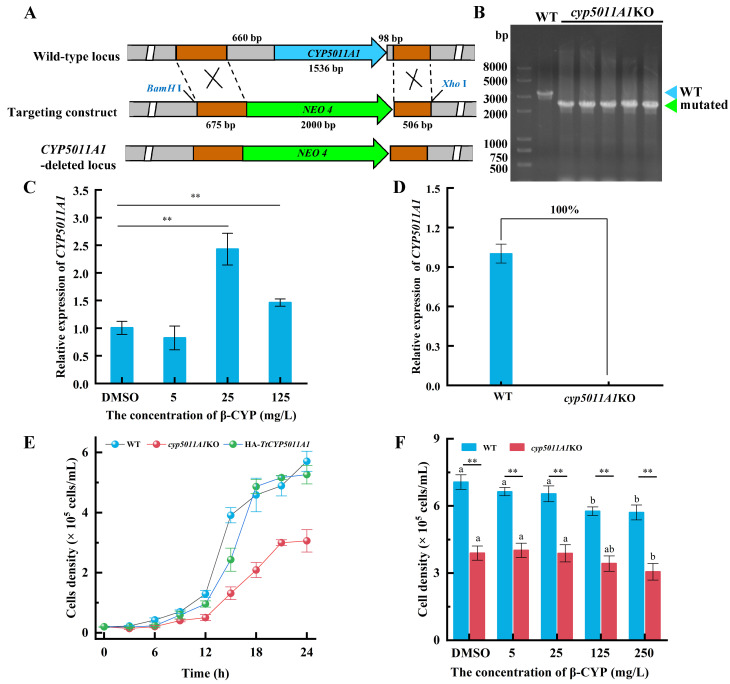
Construction of *CYP5011A1* knockout mutant strains and comparison of cell density across different strains. (**A**) Schematic diagram of *cyp5011A1*KO construction. Cyan arrow represents *CYP5011A1* (1536 bp), the green arrow represents the Neo4 cassette (approximately 2000 bp), and the brown rectangular boxes represents the flanking sequences of *CYP5011A1*. *CYP5011A1* was replaced by the Neo4 cassette through homologous recombination to generate mutants. X represents the location where homologous recombination occurs. (**B**) The identification of mutants *cyp5011A1*KO. The mutants *cyp5011A1*KO were identified by PCR. The fragment in WT is approximately 3296 bp, while the fragment in mutants is 2483 bp, as labeled by triangles. (**C**) Relative expression of *CYP5011A1* in WT under exposure to different concentrations of β-CYP. (**D**) The identification of mutants *cyp5011A1*KO by qRT-PCR. The relative expression of *CYP5011A1* is zero in mutants, confirming the successful generation of *CYP5011A1* knockout strains. (**E**) Comparison of cell density across different strains under 250 mg/L β-CYP stress. (**F**) Comparison of cell density across different strains exposed to varying concentrations of β-CYP for 24 h. Different letters in the bar chart denote statistically significant differences between the same strains exposed to different β-CYP concentrations, as determined by post hoc multiple comparisons. Asterisk (**) denotes highly significant difference between two groups (*p* < 0.01 from two-tailed Student’s T-test).

**Figure 5:**
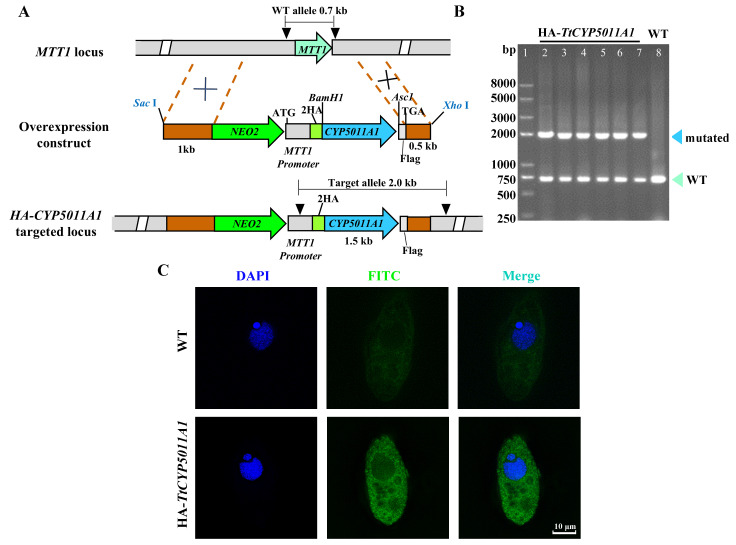
Construction of HA-*TtCYP5011A1* and immunofluorescence localization of HA-Cyp5011A1 in *T. thermophila*. (**A**) Schematic diagram of HA-*TtCYP5011A1* construction. Gene *CYP5011A1* (cyan arrow) was inserted downstream of two HA tag (2HA) under the control of MTT1 (metallothionein 1) promoter. The brown rectangular boxes represent the flanking sequences of MTT1. In *T. thermophila*, the MTT1 coding sequences in the wild-type (WT) strain were replaced by HA-*CYP5011A1* with the *NEO2* cassette through homologous recombination to generate HA-*TtCYP5011A1*. X labels the location where homologous recombination occurs. (**B**) The identification of HA-*TtCYP5011A1* by PCR. The fragment in WT is approximately 750 bp, while the fragment in the mutant is 2036 bp. (**C**) Immunofluorescence localization of HA-Cyp5011A1 in *T. thermophila*. HA-Cyp5011A1 is localized in the cytoplasm by indirect immunofluorescence via HA tagging (FITC, green). The nuclei are stained blue by DAPI.

P450 enzymes play a crucial role in the metabolism of various endogenous and exogenous compounds, catalyzing their oxidation, reduction, and hydrolysis. In *T. thermophila*, qRT-PCR analyses revealed that *CYP5011A1* was significantly upregulated in response to 25 mg/L or 125 mg/L β-CYP over 24 h (Figure 4C). The *cyp5011A1*KO mutants were constructed and identified (Figure 4A,B,D). The proliferation rate of *cyp5011A1*KO strains decreased markedly compared with that in WT (Figure 4E). GT analysis during the log-phase growth stage from 3 to 18 h revealed that *cyp5011A1*KO exhibited an extended GT of 3.92 h compared to WT’s 3.48 h. Additionally, compared to WT, the cell density of *cyp5011A1*KO strains exposed to varying concentrations of β-CYP for 24 h significantly decreased (Figure 4F). Even when exposed to DMSO, the cell density of *cyp5011A1*KO was also lower than that of WT, indicating that *CYP5011A1* is involved in the metabolism of various endogenous compounds and crucial for cellular tolerance to β-CYP stress. The observation suggests that the knockout of *CYP5011A1* increased the environmental sensitivity of *T. thermophila*, possibly due to alterations in cellular metabolism. Within cells, different P450s might have unique subcellular localizations, impacting their function and interaction with other cellular components. HA-*TtCYP5011A1* mutants were constructed and identified (Figure 5A,B). The immunofluorescence staining showed that HA-Cyp5011A1 localized in the cytoplasm (Figure 5C).

### 3.3. Amino Acids Were Primarily Changed in cyp5011A1KO Mutants

**Figure 6:**
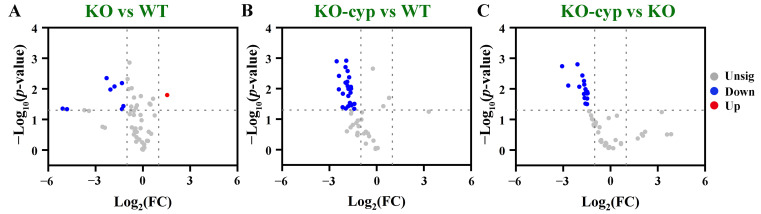
Volcano plots of metabolites’ distribution between two comparison groups. (**A**) KO vs WT; (**B**) KO-cyp vs WT; and (**C**) KO-cyp vs KO. Grey nodes (unsig), red nodes (up), and blue nodes (down) have the same meaning as in Figure 1. WT refers to wild-type cells under exposure to DMSO; KO refers to the *cyp5011A1*KO strain under exposure to DMSO; and KO-cyp refers to the *cyp5011A1*KO strain exposed to 125 mg/L β-CYP.

The metabolic profiles of *cyp5011A1*KO were analyzed using GC-MS. The PCA analysis showed that QC samples clustered together, indicating the stability and validity of the analysis (Figure S1B). A total of 51 metabolites were identified, including 9 amino acids, 8 carbohydrates, 6 fatty acyls, 3 organonitrogen compounds, and 25 other metabolites (see Metabolites’ data.xlsx in Supplementary Materials). Based on the following criteria: |log_2_(FC)| > 1 and *p*-value < 0.05 from a two-tailed Student’s T-test, compared with the WT group, 9 differential metabolites were identified in the KO group, including 8 downregulated and 1 upregulated (valine) (Figure 6A). When the *cyp5011A1*KO was exposed to β-CYP, 25 metabolites were downregulated in the KO-cyp group compared with the WT group (Figure 6B). Additionally, 17 metabolites were downregulated in the KO-cyp compared with the KO group (Figure 6C). The results indicated that *CYP5011A1* knockout affected the metabolic profiles in *T. thermophila*, particularly when *cyp5011A1*KO was exposed to β-CYP, with a larger number of metabolite levels decreased. As observed in the PCA model (Figure S1B), the KO-cyp group was clearly separated from the WT group, while the KO group partially overlapped with either the WT group or the KO-cyp group being within 95% of Hotelling’s T2 ellipse. The first two principal components explained a cumulative variance of 71.4%, with PC1 accounting for 50.2% and PC2 accounting for 21.2% of the total variance, respectively.

In the OPLS-DA models (Figure S4), similar results were observed, with the KO group partially overlapping with the WT group in the score plot. The R^2^Y and Q^2^ values for the KO vs WT model were 0.589 and 0.475, respectively. However, permutation tests (1000 iterations) for this model produced *p*-values of 0.098 for Q^2^ and 0.322 for R^2^Y, both exceeding the threshold of 0.05 (Figure S3D), suggesting potential limitations in model reliability and a risk of false positive results. To address this, the model was refined by filtering key variables using a VIP threshold (VIP > 0.8) and removing noise variables. The optimized OPLS-DA model for KO vs WT demonstrated improved performance, with R^2^Y and Q^2^ values of 0.609 and 0.550, respectively (Figure S5). Permutation tests for the optimized model yielded significant *p*-values of 0.017 for Q^2^ and 0.035 for R^2^Y, confirming its robust predictive capability and goodness of fit. Furthermore, clear separation was achieved in the other two comparison groups. The OPLS-DA model for KO-cyp vs WT exhibited R^2^Y and Q^2^ values of 0.651 and 0.413, respectively, while the model for KO-cyp vs KO showed R^2^Y and Q^2^ values of 0.587 and 0.479, respectively (Figure S4). The Q^2^ values were greater than 0.4, and the *p*-values for the permutation test were less than 0.05, indicating strong explanatory power and good predictability of the models (Figure S3).

In a comprehensive analysis of the differential metabolites in the three comparison groups, based on a VIP value greater than 1.0, 31 SCMs were identified after removing duplicate metabolites (Figure S6). Five differential metabolites were identified from KO vs WT, 25 from KO-cyp vs WT, and 17 from KO-cyp vs KO, respectively (Table S3 and Figure S7A). The changes in these SCMs across the comparison groups are shown in the Venn diagram and no common metabolites were altered across all three comparison groups. Isoleucine, amphetamine, and serine were decreased only in the KO vs WT group. Linolenic acid, pentadecanoic acid, D-lactose monohydrate, palmitic acid, oleic acid, phosphoric acid, myristic acid, malic acid, glycine, and 9,12-octadecadienoic acid were decreased only in the KO-cyp vs WT group. Valine was altered in both KO vs WT and KO-cyp vs KO groups, with levels increasing in the KO group and decreasing in the KO-cyp group. However, threonine and N-acetyl glucosamine methoxime were decreased only in the KO-cyp vs KO group. The classification of these SCMs revealed that amino acids, fatty acids, carbohydrates, and organonitrogen compounds were primarily altered, with amino acids accounting for 16.1% of the total SCMs, including serine, isoleucine, tyrosine, glycine, valine, and threonine (Figure S7B). This suggests that the knockout of *CYP5011A1* primarily affects amino acid-related metabolic pathways.

### 3.4. CYP5011A1 Affects Five Metabolism Pathways

**Figure 7:**
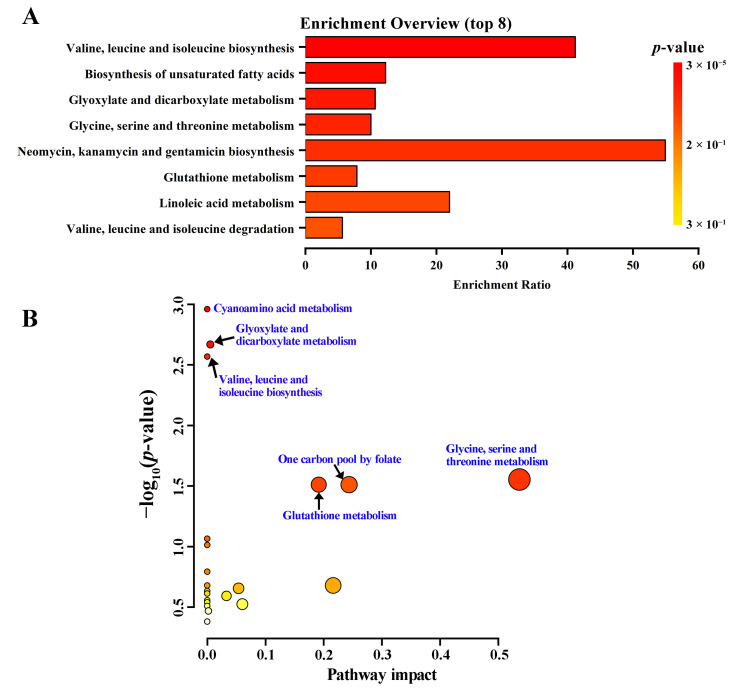
Enrichment analysis and pathway analysis of differential metabolites in *cyp5011A1*KO compared with WT. (**A**) The enrichment analysis (top 8, *p*-values < 0.05). Yellow and red indicate high and low *p*-values, respectively. The horizontal axis represents enrichment ratio with larger values indicating a greater number of metabolites annotated to the pathway. (**B**) Summary of pathway analysis. Each bubble represents a specific pathway. Bubble size increases with pathway impact values, and colors indicate −log_10_*p*-values, with darker colors representing more critical metabolic pathways. Pathways with *p*-values < 0.05 are considered significantly enriched and are labeled with blue text.

**Figure 8:**
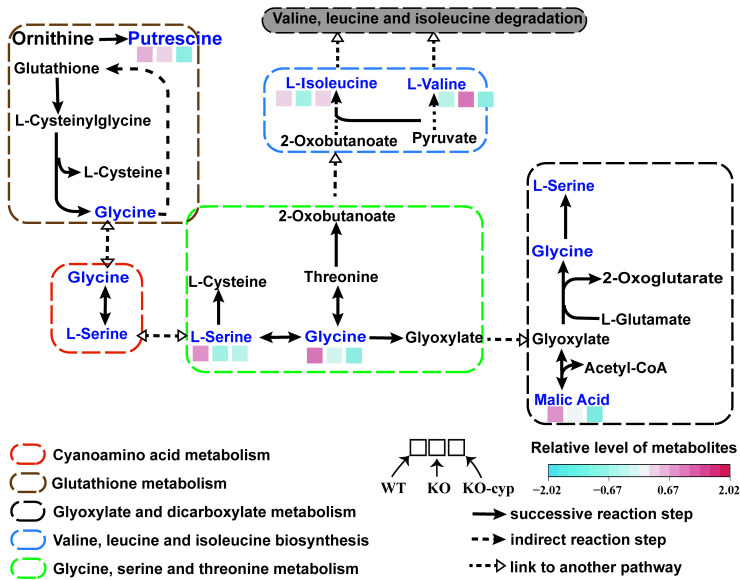
Schematic diagram of related metabolic pathways in *cyp5011A1*KO compared with WT. Differential metabolites are marked in blue text with colored square box in schematic diagram. The colors within the square boxes indicate the metabolites’ level in cells. Arrows represent the direction of reactions. Dotted black solid arrows indicate metabolites that indirectly participate in reaction steps, while dashed black hollow arrows indicate metabolites involved in other metabolic pathways. Dotted rectangles with rounded corners represent the metabolic pathways.

KEGG analysis identified eight significantly altered metabolism pathways (Figure 7A). Pathway analysis revealed that six metabolism pathways were significantly changed, including cyanoamino acid metabolism, glyoxylate and dicarboxylate metabolism, valine–leucine and isoleucine biosynthesis, glycine–serine and threonine metabolism, glutathione metabolism, and one carbon pool by folate (Figure 7B and Table 2). Combining the results of these two analyses, it was concluded that the knockout of *CYP5011A1* significantly affects glyoxylate and dicarboxylate metabolism, as well as amino acid metabolisms, including valine–leucine and isoleucine biosynthesis, glycine–serine and threonine metabolism, and glutathione metabolism. The SCMs identified in these four metabolic pathways primarily include amino acids (glycine, serine, isoleucine, and valine), hydroxy acids (malic acid), and organonitrogen compounds (putrescine). Compared to the WT group, valine was significantly upregulated, while serine and isoleucine were downregulated in the KO group (Figure 8 and Figure S8). When *cyp5011A1*KO was exposed to β-CYP, glycine, malic acid, and putrescine were remarkedly downregulated. Compared to the KO group, valine, and putrescine levels were significantly downregulated in the KO-cyp group. These observations suggest that the knockout of *CYP5011A1* affects the biosynthesis of valine, leucine, and isoleucine in *Tetrahymena*, further disrupting metabolic pathways related to glycine and serine upon exposure to the insecticide β-CYP.

Given all of these findings, *CYP5011A1* is crucial for *T. thermophila* to tolerate high concentrations of β-CYP. While *T. thermophila* can enhance its tolerance to β-CYP by regulating carbohydrate metabolisms, the knockout of *CYP5011A1* indirectly affected the amino acid metabolism, leading to a decreased proliferation rate of *T. thermophila* exposed to β-CYP and altered environmental sensitivity.

## 4. Discussions

In response to abiotic stress, numerous pathways associated with the cellular carbohydrate metabolism are modulated to maintain energy homeostasis. Starch and sucrose metabolism, a crucial source of glucose, fulfills increased energy demands during carbohydrate catabolism. Starch and sucrose metabolism changes have been observed in the gills of *Symphysodon aequifasciatus* under cold stress [24] and in the brackish water flea *Diaphanosoma celebensis* exposed to methylmercury [25]. Luteolin protects against glutamate-induced PC12 cell injury by modulating starch and sucrose metabolism, among other mechanisms [26]. In *T. thermophila*, exposure to 125 mg/L β-CYP induces significant changes in starch and sucrose metabolism, notably decreasing D-fructose levels and increasing trehalose and maltose levels (Figure 3C). Trehalose can be enzymatically hydrolyzed to maltose, which is further metabolized into D-glucose, while D-fructose can also be converted into D-glucose, thereby contributing to cellular energy homeostasis. This implies an energy deficit in *T. thermophila* under β-CYP stress. Beyond replenishing intracellular energy, oligosaccharides also play various biological roles, including osmoprotection, regulatory functions, signaling for symbiosis, and antioxidant activity to mitigate the impact of stressful conditions [27]. Trehalose, a nonreducing disaccharide, serves as a good stress protector for cell membranes and cellular proteins by hindering protein aggregation and degradation via promoting the formation of phosphate groups and hydrogen bonds, and as an osmoprotectant for adjustment of the osmotic environment of cells [28]. The elevated trehalose concentrations protect cells from thermal stress [29], and ethanol stress [30]. Thus, increasing trehalose levels may enhance the adaptation of *T. thermophila* to 125 mg/L β-CYP. However, increased intracellular trehalose synthesis also imposes a metabolic burden, which can impede cell growth and delay the cell cycle [31].

Galactose metabolism is essential for balancing glucose levels in organisms, as it is involved in the conversion of galactose to glucose derivatives such as α-D-galactose and glucose-6-phosphate. These processes are critical for the synthesis of amino sugar and nucleotide sugar, which assist in the creation of generic material, protein, and other metabolisms [32]. Disruption of galactose metabolism can lead to metabolic disorders, oxidative stress-induced cellular damage, and inhibited cell growth [33,34]. Under β-CYP stress, glycerol and myo-inositol levels are significantly increased, while fructose level decreases in *T. thermophila*. Both fructose and glycerol are important for energy production under abiotic stress. Glycerol, as a key intermediate in energy metabolism, is involved in phospholipid biosynthesis, which is necessary for cellular membranes [35] and can also generate ATP through substrate-level phosphorylation [36]. Upon an increase in external osmolarity, the accumulation of glycerol content within cells helps protect against high osmolarity pressure [37]. Thus, elevated glycerol biosynthesis serves as a protective agent in the response to environmental stressors, such as ethanol, by correspondingly consuming reducing NAD(P)H and thereby balancing redox potential [38]. Inositol is a prevalent carbocyclic sugar polyalcohol that exists in nine stereoisomers due to the configuration of hydroxyl groups, and it functions as a critical metabolic regulator. In *Tetrahymena*, inositol isomers are present, with myo-inositol being the most significant, alongside scyllo-, chiro-, and neo-inositols [39]. Myo-inositol participates in signal transduction, glucose homeostasis modulation, ion-channel permeability regulation, stress responses, and cellular homeostasis [40,41]. It also contributes to the synthesis of phosphatidylinositols, which are essential membrane structural lipids involved in processes such as exo- and endocytosis, membrane homeostasis, and cell viability [42]. Deletion of *PRO1* encoding inositol-3-phosphate synthase results in increased sensitivity of *S. cerevisiae* to acetic acid and phenol. Conversely, overexpression of *PRO1* or supplementation with myo-inositol in the medium enhances yeast’s tolerance to these stresses [43]. Similar results are observed in HEK293T cells [44]. Thus, the significant increase in myo-inositol levels in *T. thermophila* under β-CYP stress may enhance its tolerance to β-CYP stress. Additionally, an increase in the L-Chiro-Inositol level was also observed in the 125 mg/L β-CYP treatment group (Figure 2C). Myo-inositol enters cells via sodium ion- and proton-dependent transport and can be converted into chiro-inositol via a NADH/NADPH-dependent epimerase in the cytosol or on the membrane during metabolic stress [40]. Therefore, this compound may also play significant roles in *T. thermophila* under cypermethrin stress.

The cyanoamino acid metabolism pathway, which involves the chemical reactions of cyanoamino acids or amino acid derivatives containing a cyanide group, plays a vital role in nitrogen metabolism. When *Bactrocera dorsalis* larvae are exposed to botanical insecticides such as azadirachtin or hormone analogs like pyriproxyfen, cyanoamino acid metabolism is notably impacted [45]. Pesticide atrazine significantly increases the concentration of L-asparagine and other amino acids in mycoinsecticide *Metarhizium robertsii* [46]. Asparagine not only serves as a substrate for protein synthesis but also provides carbon and nitrogen for nucleotide synthesis [47]. It acts as an amino acid exchange factor, regulating the uptake of extracellular amino acids to balance protein and nucleotide synthesis, thus promoting cell proliferation [48]. As a non-essential amino acid, asparagine can be synthesized in vivo or obtained from dietary sources, with its metabolism regulated primarily by asparagine synthase (ASNS) and asparaginase [49]. Knockdown of ASNS suppresses the proliferation of human melanoma cells [50], while the D330V mutation in ASN1 (asparagine synthase 1) in *Saccharomyces cerevisiae* with ASN2 knockout impedes cell growth [51]. This indicates the essential role of asparagine in cell growth, with decreased levels leading to slower cell proliferation. Additionally, asparagine alleviates osmotic pressure, as demonstrated by how supplementing asparagine in the medium mitigates the stress effects of 1.5% NaCl on *Methylocystis sp.* strain SC2 as asparagine effects proteome rearrangements and metabolic pathway activity changes [52].

To explore whether the β-CYP-induced gene *CYP5011A1* is involved in β-CYP detoxification, we constructed *cyp5011A1*KO mutants, which showed a proliferation rate decreased compared with WT. This is consistent with findings in other organisms [13,14]. Further analysis of metabolomic profiling in *cyp5011A1*KO revealed significant alterations in several pathways associated with amino acid metabolism. Consistent with these findings, silencing *CYP307A1* primarily disrupted amino acids and their metabolites in the midguts of 4th instar larvae [53]. Valine, leucine, isoleucine, phenylalanine, and tyrosine are highly correlated with both Cyp1A2 activity and gene expression in rats under β-naphthoflavone stress [54].

Amino acids play crucial roles in many metabolic pathways, acting both as key substrates and regulators. They are involved in repairing the antioxidant system to maintain the cellular antioxidant balance, preventing the oxidative stress damage induced by pesticides [55], and balancing osmotic pressure inside and outside of the cells to protect cells from external stresses [56]. In a previous study [18], we observed that β-CYP treatment triggered oxidative stress responses in *Tetrahymena*. In the present study, four differential amino acid metabolites (glycine, serine, isoleucine, and valine) were observed to participate in significantly changed metabolic pathways.

Branched-chain amino acids, including valine, leucine, and isoleucine, act not only as signaling molecules but also as potential energy sources through their metabolism of glutamate and acetyl-CoA [57]. In addition, branched amino acids alone or in combination have been demonstrated to mediate the antioxidant defense system, thereby reducing oxidative stress by lowering mitochondrial reactive oxygen species [58,59]. However, elevated branched amino acid levels exert deleterious effects, which are linked to increased ROS in cells [60]. Consequently, exposure to insecticides often disrupts the metabolic pathways of branched-chain amino acids. For instance, sublethal doses of chlorpyrifos upregulated valine and isoleucine biosynthesis in HepG2 cells [61]. EC_50_ exposure to chlorpyrifos has been reported to downregulate isoleucine levels in *Caenorhabditis elegans* [62]. Neonicotinoids such as dinotefuran, nitenpyram, and acetamiprid have been shown to interfere with valine–leucine and isoleucine biosynthesis in ICR mice livers, with significant increases in these branched-chain amino acids, contributing to lipid accumulation and oxidative stress [63]. Serine metabolism is linked to nucleotide and lipid metabolism, and one-carbon unit metabolism. It has been found that serine, as a central node linking glycolysis to glutathione biosynthesis [64], is used as a nutritional supplement in stress conditions to maintain metabolic homeostasis and provide essential substrates for cell proliferation [65]. Serine deficiency aggravated oxidative stress, mitochondrial dysfunction, apoptosis, and proliferation inhibition in IPEC-J2 cells, while serine supplementation alleviated redox imbalance and proliferation defects [66]. In diquat-induced mice and hepatocytes, serine supplementation also alleviated oxidative stress by enhancing glutathione synthesis [64]. Glycine, primarily synthesized from serine, has been reported to scavenge oxygen-free radicals [67] and promote the synthesis of glutathione [68], thereby protecting cells from oxidative damage and apoptosis, ultimately reducing cell mortality [69]. Exogenous toxicants are known to induce oxidative stress and apoptosis in organisms, often disrupting metabolic pathways involving glycine and serine. For instance, in *Chlorella vulgaris* exposed to atrazine-desethyl, the most significantly downregulated pathways included glycine–serine and threonine metabolism, as well as glyoxylate and dicarboxylate metabolism with decreased glycine and serine levels [70]. *CYP5011A1* knockout led to decreased isoleucine and serine levels, but increased valine levels in *Tetrahymena* (Figure S7), which implies that *CYP5011A1* knockout is subject to oxidative stress. Meanwhile, exposure to β-CYP further decreased the glycine level in the mutant strain, confirming *cyp5011A1*KO is more susceptible to β-CYP than the wild-type. This suggests that Cyp5011A1 plays a critical role in balancing the antioxidant defense system, likely through the indirect regulation of branched-chain amino acids’ biosynthesis and glycine–serine and threonine metabolism in *Tetrahymena*. The detailed mechanism of how *CYP5011A1* acts in amino acid metabolisms needs further investigation.

Putrescine and spermidine, the predominant polyamines in *Tetrahymena* [71], are essential for normal cellular proliferation and differentiation [72]. In *Tetrahymena*, putrescine biosynthesis from ornithine is likely the only major route during exponential growth [72]. Polyamines, including putrescine, can bind to members of the cytochrome P450 family, playing a role in the detoxification of external toxic substances [73]. Compared to the WT, the putrescine level in the *cyp5011A1*KO mutants exposed to β-CYP was significantly reduced. Notably, glycine and serine are key junctions in several critical metabolic pathways, including cyanoamino acid metabolism, glyoxylate and dicarboxylate metabolism, and glutathione metabolism (Figure 8). The significant alterations observed in glycine and serine levels in the present study disrupt these interconnected pathways, supporting their central role in cellular metabolic regulation in *Tetrahymena*. Malic acid was also significantly decreased in the KO-cyp group compared with the WT group. Malic acid, glycine, and serine are involved in glyoxylate and dicarboxylate metabolism, which plays a crucial role in carbon and energy metabolism in certain invertebrate cells, contributing to the regulation of carbon and energy supply [74]. The primary function of the glyoxylate cycle is to support growth when only two-carbon substrates are available, thereby facilitating the growth of protozoa [75]. Treatment with desmethylimipramine inhibited the glyoxalate cycle in *Tetrahymena* [76]. In response to perfluorooctanoic acid treatment, proteins involved in glyoxylate and dicarboxylate metabolism in *E. coli* were upregulated, suggesting a potential mechanism of resistance to perfluorooctanoic acid [77]. In the present study, *cyp5011A1* knockout led to the downregulation of serine levels. When *cyp5011A1*KO was exposed to β-CYP, glycine and malic acid levels were downregulated, suggesting a downregulation of glyoxylate and dicarboxylate metabolism in *Tetrahymena*. These findings also imply that knockout of *cyp5011A1* resulted in the higher toxicity of β-CYP to *Tetrahymena*. These findings would offer important insights for formulating strategies to reduce the effects of pesticides on non-target organisms and ecosystems.

## 5. Conclusions

*T. thermophila* tolerates high levels of β-CYP stress. In the present study, untargeted metabolomics analysis of *T. thermophila* revealed significant perturbations in three major metabolic pathways: starch and sucrose metabolism, galactose metabolism, and cyanoamino acid metabolism. Specifically, metabolites associated with carbohydrate metabolism including trehalose, maltose, glycerol, and D-myo-inositol were upregulated in response to β-CYP exposure. The transcriptional level of *CYP5011A1* was upregulated in *T. thermophila* exposed to β-CYP. Knockout of *CYP5011A1* led to a reduced proliferation rate under β-CYP stress. The metabolomics analysis in *cyp5011A1*KO showed that amino acid metabolism, including valine–leucine and isoleucine biosynthesis and glycine–serine and threonine metabolism, was significantly altered, with significantly changed amino acids such as serine, isoleucine, and valine. When *cyp5011A1*KO mutants were exposed to β-CYP, glutathione metabolism and glyoxylate and dicarboxylate metabolism were downregulated, with the levels of glycine, malic acid, and putrescine being decreased compared with the WT group, suggesting that the knockout of *cyp5011A1* affected amino acid metabolisms. These findings confirmed that *T. thermophila* develops β-CYP tolerance through carbohydrate metabolism reprogramming, Cyp5011A1 upregulation, and altered amino acid metabolisms.

## Figures and Tables

**Table 1:** Perturbed metabolic pathways in *T. thermophila* under exposure to β-CYP, compared with CK.

Pathway Name	Total	Hits	Raw *p*-Value	Impact	Match Status
Starch and sucrose metabolism	16	3	0.001	0.163	D-Fructose, alpha-Trehalose, Maltose
Galactose metabolism	24	3	0.002	0.000	D-Fructose, Glycerol, Myo-Inositol
Cyanoamino acid metabolism	4	1	0.046	0.000	L-Asparagine
alpha-Linolenic acid metabolism	12	1	0.131	0.000	Linolenic acid
Fructose and mannose metabolism	14	1	0.152	0.000	D-Fructose
Alanine, aspartate and glutamate metabolism	14	1	0.152	0.000	L-Asparagine
Glycerolipid metabolism	14	1	0.152	0.082	Glycerol
Inositol phosphate metabolism	21	1	0.220	0.000	Myo-Inositol
Amino sugar and nucleotide sugar metabolism	27	1	0.274	0.000	D-Fructose
Purine metabolism	46	1	0.425	0.000	Adenosine

Notes: total is the total number of metabolites in the pathway; hits, the actually matched number from the user differential metabolites’ data; and raw *p*-value, the original *p*-value calculated from the enrichment analysis by using MetaboAnalyst 6.0. Impact, the pathway impact value obtained from topology measure by using MetaboAnalyst 6.0. Match status, differential metabolites hitted.

**Table 2:** Perturbed metabolic pathways in cyp5011A1KO compared with WT.

Pathway Name	Total	Hits	Raw *p*-Value	Impact	Match Status
Cyanoamino acid metabolism	4	2	0.001	0.000	Glycine; L-Serine
Glyoxylate and dicarboxylate metabolism	20	3	0.002	0.005	Malic Acid; Glycine; L-Serine
Valine, leucine and isoleucine biosynthesis	6	2	0.003	0.000	L-Isoleucine; L-Valine
Glycine, serine and threonine metabolism	19	2	0.028	0.535	Glycine; L-Serine
Glutathione metabolism	20	2	0.031	0.191	Glycine; Putrescine
One carbon pool by folate	20	2	0.031	0.243	Glycine; L-Serine
Valine, leucine and isoleucine degradation	35	2	0.086	0.000	L-Isoleucine; L-Valine
Arginine biosynthesis	7	1	0.097	0.000	Urea
alpha-Linolenic acid metabolism	12	1	0.161	0.000	(9Z,12Z,15Z)-Octadecatrienoic Acid
Arginine and proline metabolism	16	1	0.209	0.000	Putrescine
Starch and sucrose metabolism	16	1	0.209	0.216	D-Glucose
Pyruvate metabolism	17	1	0.221	0.054	Malic Acid
Pantothenate and CoA biosynthesis	18	1	0.232	0.000	L-Valine
Carbon fixation by Calvin cycle	19	1	0.243	0.000	Malic Acid
Citrate cycle (TCA cycle)	20	1	0.255	0.033	Malic Acid
Fatty acid elongation	22	1	0.277	0.000	Palmitic Acid
Fatty acid biosynthesis	23	1	0.287	0.000	Palmitic Acid
Galactose metabolism	24	1	0.298	0.060	D-Glucose
Cysteine and methionine metabolism	25	1	0.308	0.000	L-Serine
Lipoic acid metabolism	28	1	0.339	0.002	Glycine
Fatty acid degradation	36	1	0.415	0.000	Palmitic Acid

## Data Availability

All relevant data are within the paper and its additional files. The data used to support the findings of this study are available upon reasonable request.

## Supplementary Materials

The following supporting information can be downloaded at https://www.mdpi.com/article/10.3390/metabo15030143/s1: Figure S1: The score plot of PCA analysis; Figure S2. OPLS-DA score plot and model overview of the OPLS-DA model between two comparison groups; Figure S3. Permutation test of the OPLS-DA model (1000 iterations); Figure S4. OPLS-DA score plot and model overview of the OPLS-DA model between two comparison groups; Figure S5. The optimized OPLS-DA model for KO_vs_WT; Figure S6. The hierarchically clustered heatmap of SCMs in KO-cyp, KO, and WT; Figure S7. Venn diagram and pie chart of classes of differential metabolites from *cyp5011A1*KO compared with WT; Figure S8. Relative concentration of differential metabolites in KO-cyp, KO, and WT; Table S1. Primer sequences used in the assay; Table S2. Differential metabolites between any two groups; Table S3. Differential metabolites between any two groups.
