# Supplementary material for: Metabolomics’ Change Under β-Cypermethrin Stress and Detoxification Role of CYP5011A1 in Tetrahymena thermophila
Source: Metabolites. 2025 Feb 20;15(3):143. doi: 10.3390/metabo15030143 (PMC11944115; doi:10.3390/metabo15030143)
Supplement: Supplementary file 1 [file metabolites-15-00143-s001.zip › Supplementary data.pdf]

Supporting Information

Metabolomics Change Under β-Cypermethrin Stress and Detoxification Role of *CYP5011A1* in *Tetrahymena thermophila*

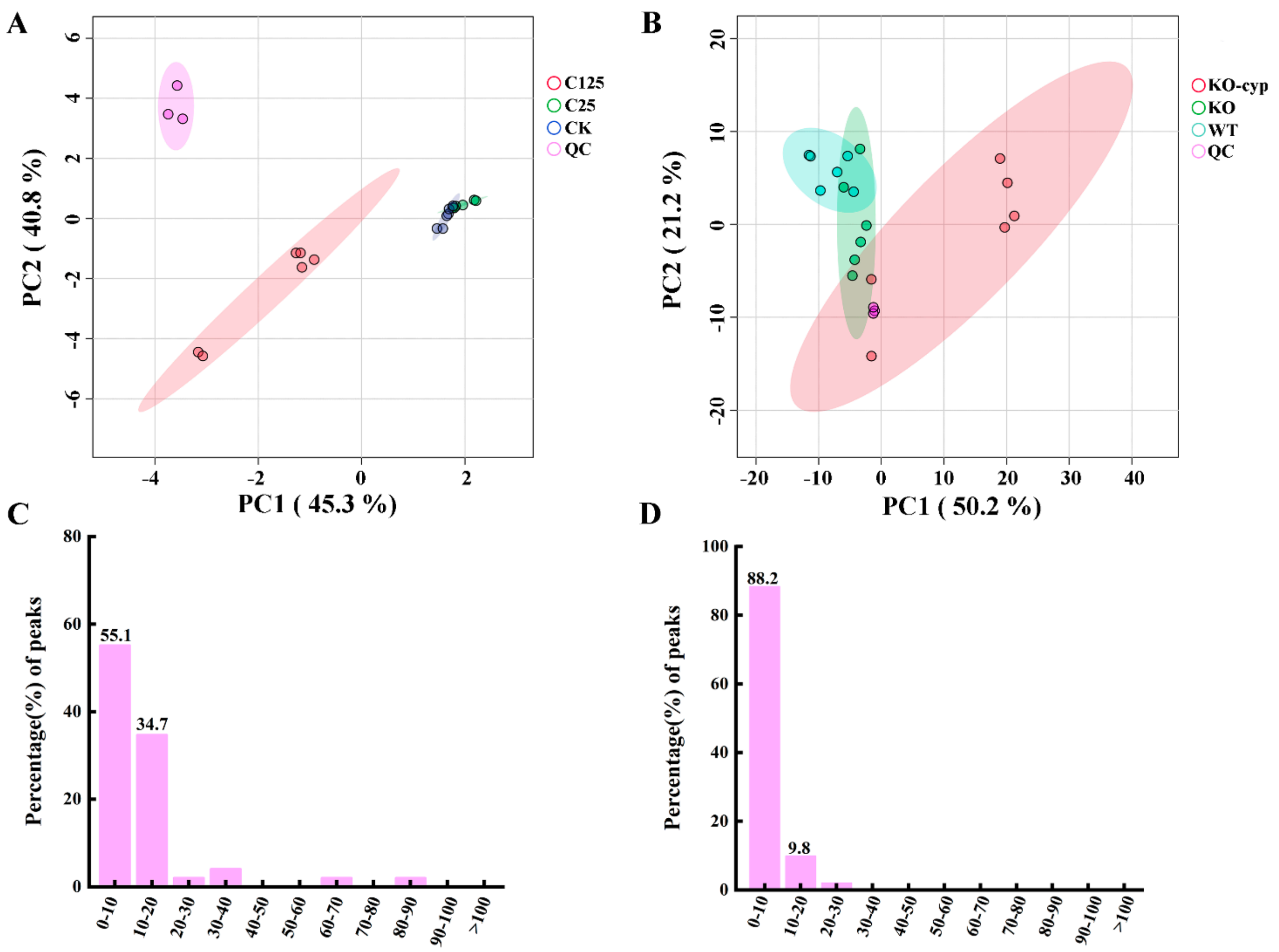

Figure_S1 The score plot of PCA analysis. (A) PCA score plot of wild-type cells exposed to different concentrations of β-CYP, PC1 (45.3%), principal component 1 accounted for 45.3% of the total variance; PC2 (40.8%), principal component 2 accounted for 40.8% of total variance; (B) PCA score plot of *cyp5011A1*KO compared with WT. The ellipse represents Hotelling 95% T2 ellipse, and all sample points are within a 95% confidence interval. (C) Coefficient of variation distribution of QC samples in Figure S1A. The abscissa represents the percentage range of the calculated coefficient of variation values, while the ordinate denotes the percentage of the total number of peaks that fall within the corresponding coefficient of variation value range. (D) Coefficient of variation distribution of QC samples in Figure S1B. Coefficient of variation = (Standard deviation of peak area / Mean peak area)×100%.

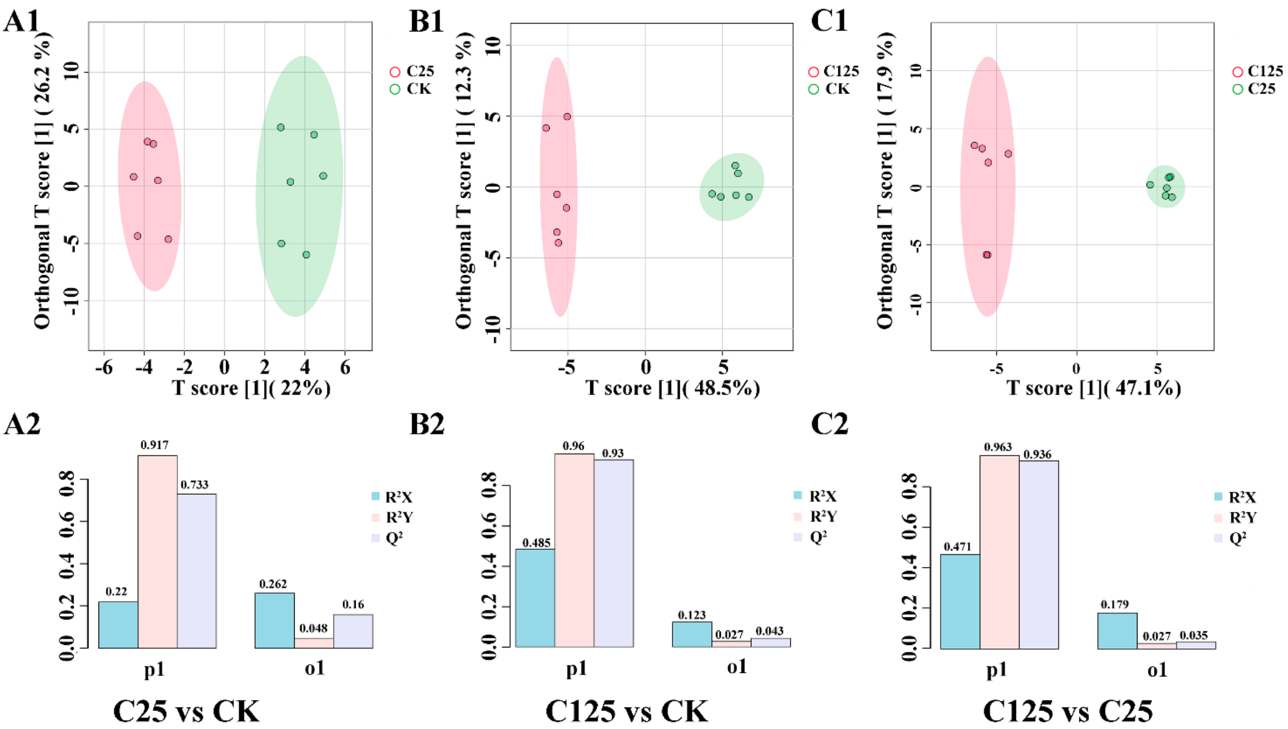

Figure S2. OPLS-DA score plot and model overview of the OPLS-DA model between two comparison groups. (A) C25 vs CK. A1, OPLS-DA score plot in C25 vs CK. it shows the separation of the C25 group from the CK group, with the ellipse representing the Hotelling 95% T2 ellipse; A2, the cross-validated R^2Y and Q^2 valuse obtained from the OPLS-DA model were 0.917 and 0.733, respectively. (B) C125 vs CK. B1, OPLS-DA score plot in C125 vs CK. It shows showing the separation of the C125 group from the CK group; B2, the cross-validated R^2Y and Q^2 valuse obtained from the OPLS-DA model were 0.96 and 0.93, respectively. (C) C125 vs C25. C1, OPLS-DA score plot in C125 vs C25. It shows the separation of the C125 group from the C25 group, C2, the cross-validated R^2Y and Q^2 valuse obtained from the OPLS-DA model were 0.963 and 0.936, respectively. R^2X: the explanation rate of the X matrices; R^2Y: the explanation rate of the Y matrices; Q^2: the prediction ability. Biological replicates are shown independently (n=6).

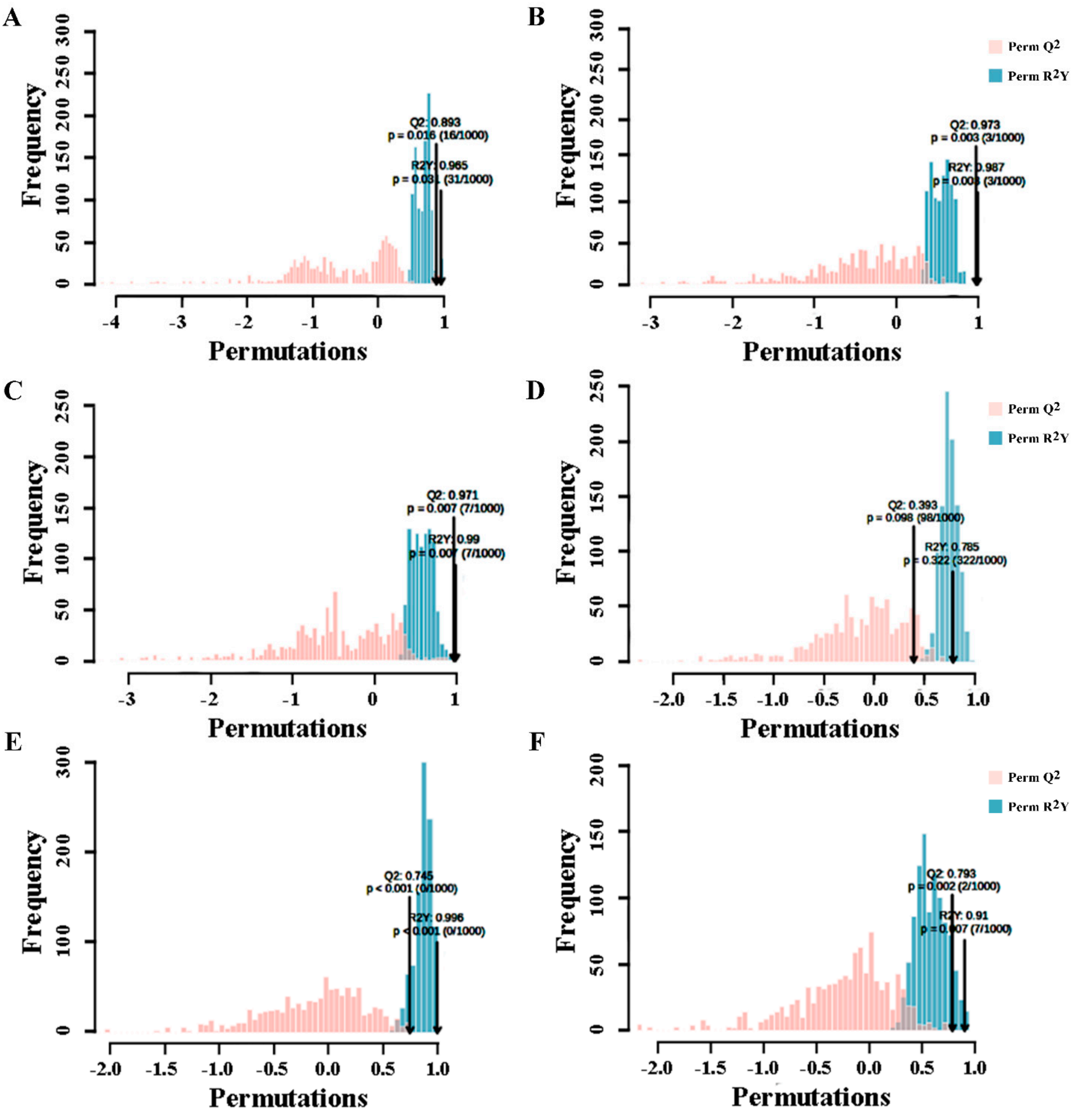

Figure S3. Permutation test of the OPLS-DA model (1000 iterations) (A) C25 vs CK; (B) C125 vs CK; (C) C125 vs C25; (D) KO vs WT; (E) KO-cyp vs WT; (F) KO-cyp vs KO. The *P*-value of the Q^2 value obtained from the 1000 iterations permutation test was less than 0.05, indicating that the model exhibits robust predictive ability without overfitting.

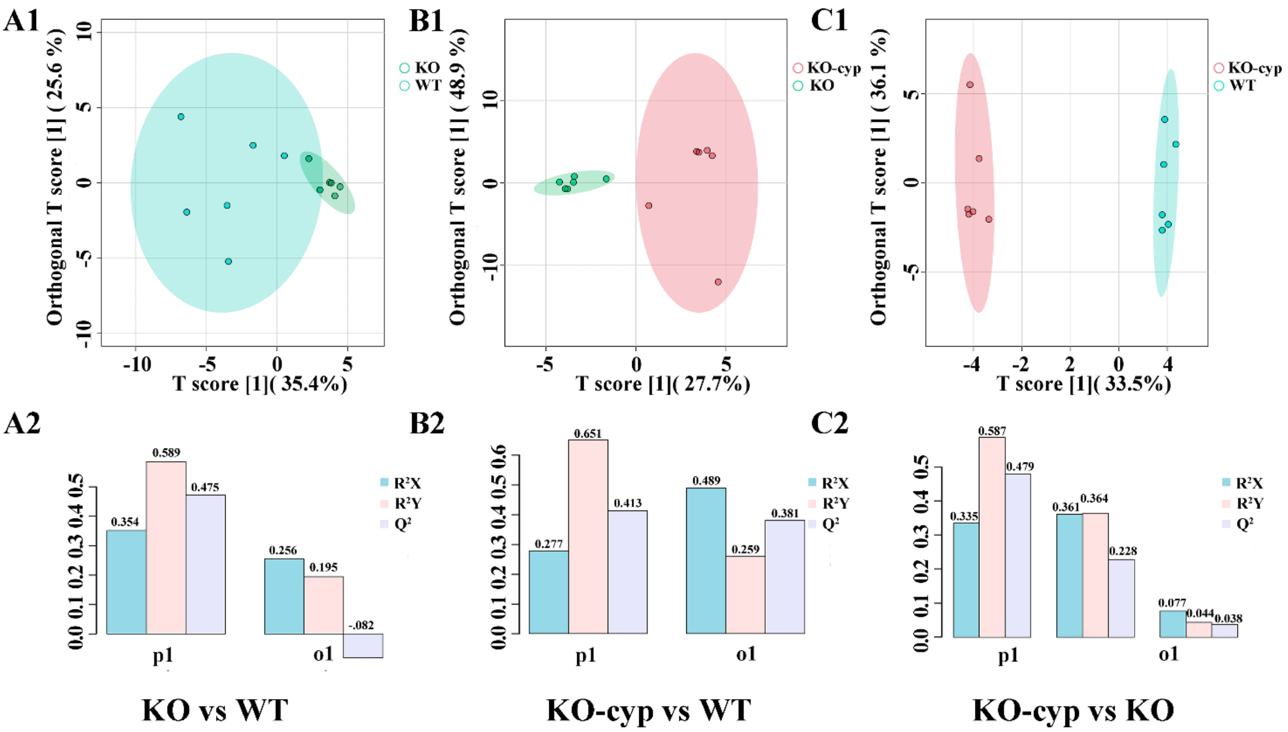

Figure S4. OPLS-DA score plot and model overview of the OPLS-DA model between two comparison groups. (A) KO vs WT. A1, OPLS-DA score plot in KO vs WT. The score plot shows the partial overlap of the KO group with the WT group, and the ellipses represent 95% confidence intervals; A2, the cross-validated R^2Y and Q^2 valuse obtained from the OPLS-DA model were 0.589 and 0.475, respectively. (B) KO-cyp vs WT. B1, OPLS-DA score plot in KO-cyp vs WT. It shows the separation of the KO-CYP group from the WT group; B2, the cross-validated R^2Y and Q^2 valuse obtained from the OPLS-DA model were 0.651 and 0.413, respectively. (C) KO-CYP vs KO. C1, OPLS-DA score plot in KO-CYP vs KO. It shows the separation of the KO-CYP group from the KO group; C2, the cross-validated R^2Y and Q^2 valuse obtained from the OPLS-DA model were 0.587 and 0.479, respectively. Biological replicates are shown independently (n=6).

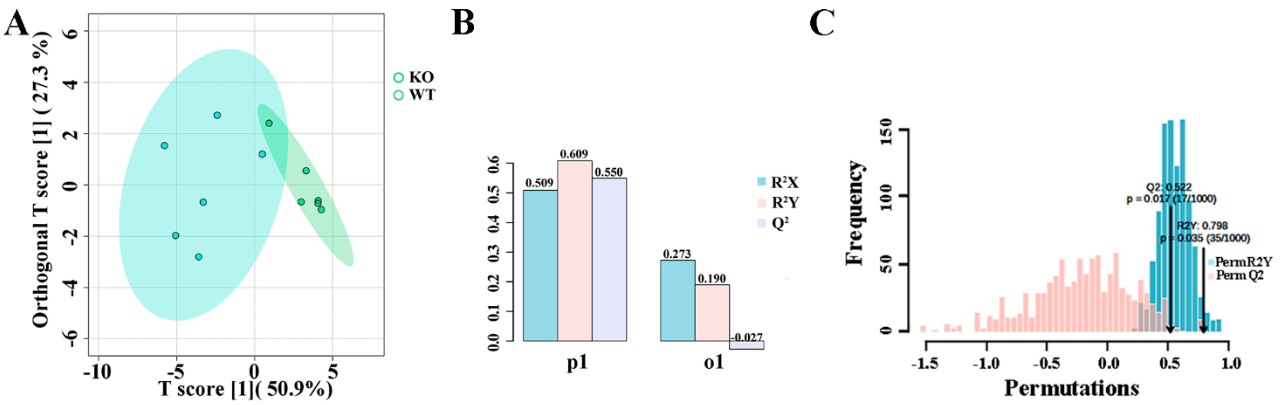

Figure S5. **The optimized OPLS-DA model for KO_vs_WT**. (A) OPLS-DA score plot. (B) The cross-validated R^2Y and Q^2 valuse derived from the optimized OPLS-DA model were 0.509 and 0.609, respectively. (C) Permutation test of the optimized OPLS-DA model (1000 iterations). The *P*-value of 0.017 for Q^2 value derived from the permutation test (1000 iterations) was less than 0.05, indicating that the model exhibits robust predictive ability without overfitting.

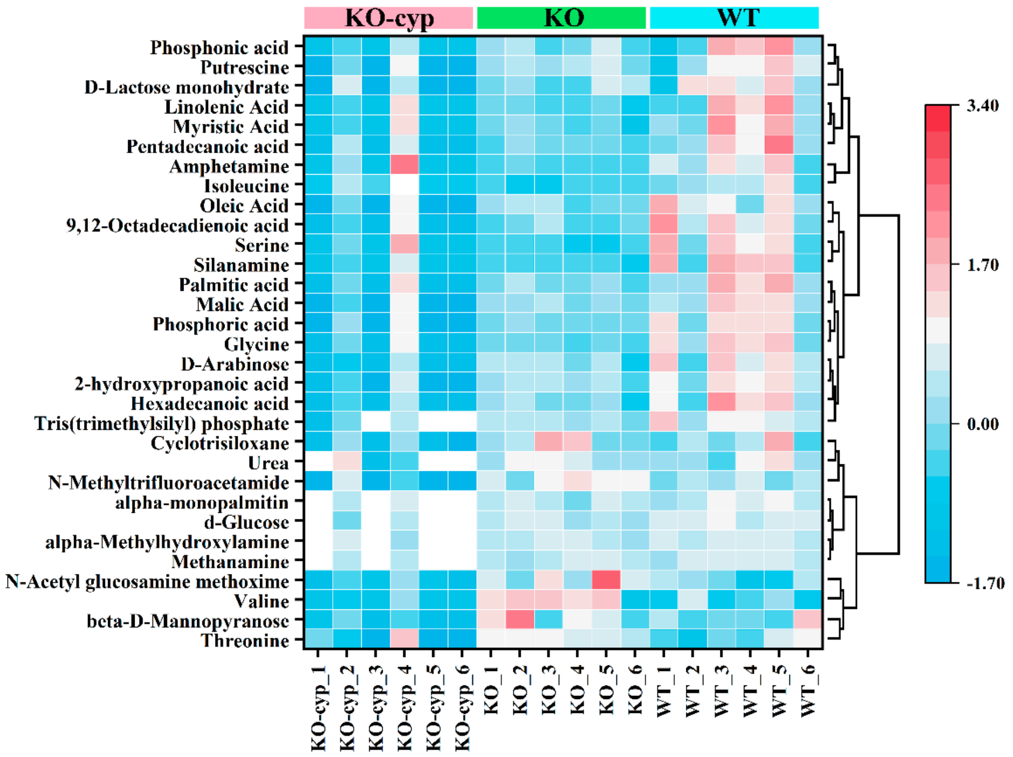

Figure S6. **The hierarchically clustered heatmap of SCMs in KO-cyp, KO, and WT**. Hierarchical clustering of 31 SCMs ($p < 0.05$, VIP > 1, $|\log_2 FC| > 1$) in KO-cyp, KO, and WT by using the normalized and pareto scaled relative metabolite data. Blue to red marks relative metabolite level from low to high.

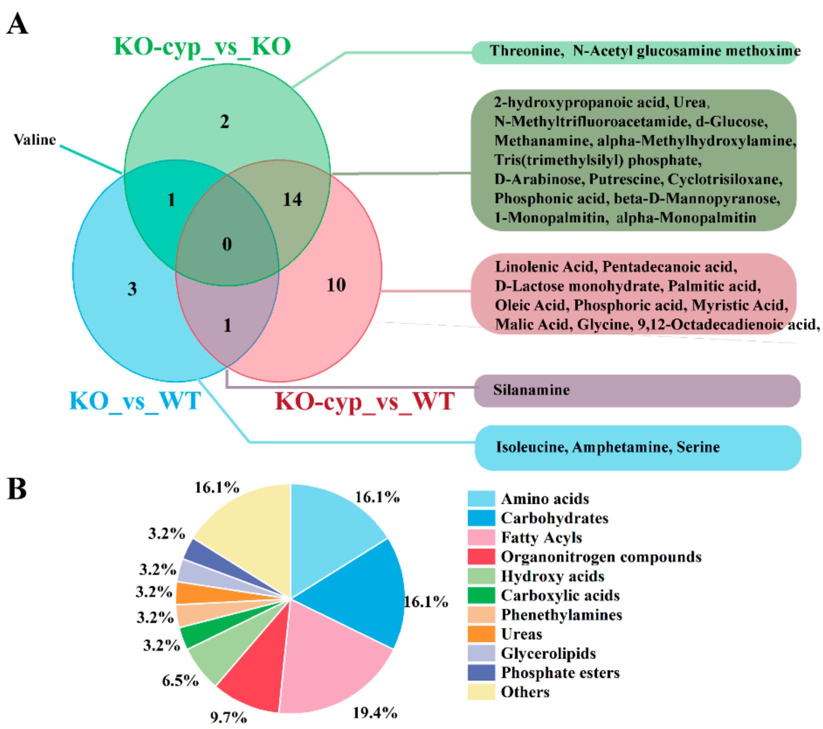

Figure S7. Venn diagram and pie chart of classes of differential metabolites from *cyp5011A1*KO compared with WT. (A) Venn diagram of 31 differential metabolites. The corresponding differential metabolites are list in the rounded rectangles. (B) The pie chart of classes of differential metabolites.

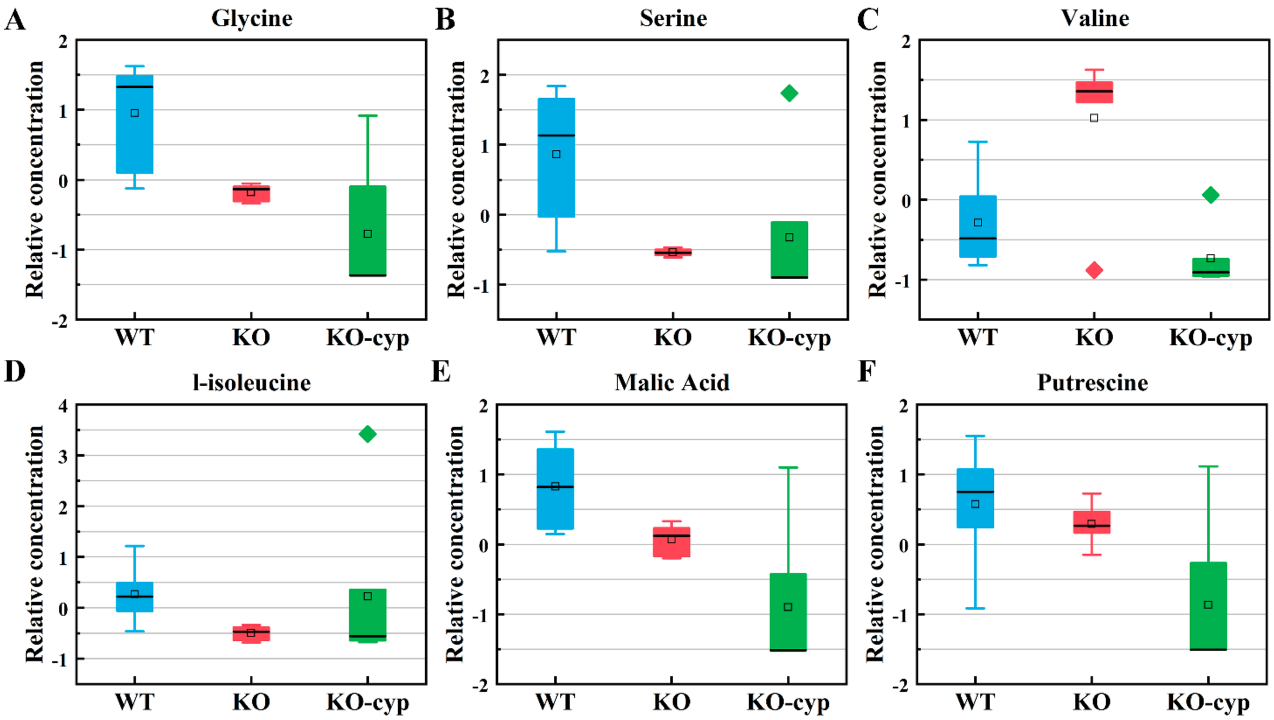

Figure S8. Relative concentration of differential metabolites in KO-cyp, KO, and WT. The graphs were ploted using the normalized and pareto scaled relative metabolites data. (A) Glycine; (B) Serine; (C) Valine; (D) l-isoleucine; (E) Malic acid; (F) Putrescine.

Table S1. Primer sequences used in the assay

GENE ID	Primers name	F sequence of primers (5'-3')	R sequence of primers (5'-3')
TTHERM_00527100	OE-*CYP5011A1*	GGATCC GTAATTTACCCTGTAGCAAACT	GGCGCGCC TCAATTTTTTTATTTTAGTCTTTCA
	KO- *CYP5011A1*-5'	CTAGAACTAGTGGATCCCACTCACTCACACACATTCT	GGTACCCGGGGGATCAATAACAAATCACCCAAATGC
	KO- *CYP5011A1*-3'	CGATACCGTCGACCTCGATCAAAGTAAATCCTTTCTCAG	CCGGGCCCCCCCTCGAGGTGAAAATATCGTCGTATCTAATTC
	q*CYP5011A1*	AAACATAGGAGGAAACATTGCT	CAAATTGTCAAAATGAAAAAGC
	JD-MTT1	GCTACGTGATTCACGATTTATGCAATG	CGAAACTGATTTTATGCAATTATGAATTAC
	JD-KO-*CYP5011A1*	ATAGATTTAAAGATATTCCAAT	GAAAGCAATATCATTCTAAC
	17s rRNA	GATCCTGCCAGTTACATATGCTTG	GCCCAACAATTAGCTCGGTTATCC

Notes: Red fonts indicated restriction sites, where GGATC and CTCGA were defective *BamH* I and *Xho* I restriction sites

Table S2 Differential metabolites between any two groups

Comparision groups	Metabolites	FC	*P-value*	VIP
C25 vs CK	L-Asparagine	2.208	0.037	1.266
C125 vs CK	2-Deoxy-6-phosphogluconolactone	9.214	0.000	1.354
	L-Chiro-Inositol	5.142	0.000	1.408
	Linolenic acid	5.021	0.001	1.180
	Myo-Inositol	3.299	0.004	1.106
	Phosphonic acid	2.713	0.000	1.380
	Glycerol	2.249	0.004	1.104
	D-(+)-Trehalose	2.233	0.000	1.394
	Maltose	2.160	0.000	1.382
	N-Acetylaspartylglutamic acid	2.022	0.001	1.205
	Glycoside	0.472	0.000	1.236
	Adenosine	0.426	0.000	1.282
	d-Fructose	0.289	0.008	1.067
C125 vs C25	2-Deoxy-6-phosphogluconolactone	14.276	0.000	1.380
	L-Chiro-Inositol	6.374	0.000	1.410
	Linolenic acid	6.341	0.001	1.249
	Myristic acid	6.185	0.014	1.057
	Myo-Inositol	3.366	0.005	1.062
	D-(+)-Trehalose	2.949	0.000	1.436
	Phosphonic acid	2.890	0.000	1.374
	Glycerol	2.482	0.002	1.132
	2-Monostearin	2.308	0.000	1.363
	beta-D-Galactopyranoside	2.145	0.018	1.041
	propane	2.107	0.001	1.163
	Maltose	2.079	0.000	1.351
	Dodecanoic acid	2.065	0.000	1.400
	aminomethane	2.060	0.000	1.388
	Glycoside	0.467	0.001	1.200
	d-Fructose	0.455	0.000	1.288

Notes: FC, fold change; *P*-value, *P*-value < 0.05 from two-tailed Student's T-test; VIP, variable importance in projection value.

Table S3 Differential metabolites between any two groups

Comparision groups	Metabolites	FC	*P-value*	VIP
KO_vs_WT	Serine	0.204	0.004	1.293
	Silanamine	0.288	0.008	1.207
	Amphetamine	0.400	0.007	1.187
	Isoleucine	0.241	0.011	1.053
	Valine	2.919	0.016	1.033
KO-cyp_vs_WT	D-Arabinose	0.173	0.001	1.414
	d-Glucose	0.264	0.001	1.408
	2-hydroxypropanoic acid	0.255	0.002	1.382
	Tris(trimethylsilyl) phosphate	0.279	0.003	1.361
	Hexadecanoic acid	0.191	0.004	1.334
	alpha-Methylhydroxylamine	0.298	0.004	1.310
	9,12-Octadecadienoic acid	0.275	0.006	1.283
	Glycine	0.255	0.006	1.279
	Malic Acid	0.265	0.007	1.266
	Phosphoric acid	0.281	0.008	1.246
	methanamine	0.323	0.009	1.235
	Silanamine	0.190	0.011	1.232
	Oleic Acid	0.290	0.010	1.223
	alpha-monopalmitin	0.326	0.010	1.220
	N-Methyltrifluoroacetamide	0.316	0.014	1.183
	beta-D-Mannopyranose	0.227	0.015	1.165
	Palmitic acid	0.288	0.017	1.159
	Putrescine	0.309	0.029	1.072
	D-Lactose monohydrate	0.322	0.032	1.067
	Pentadecanoic acid	0.330	0.035	1.063
	Urea	0.378	0.032	1.060
	Myristic Acid	0.286	0.038	1.048
	Phosphonic acid	0.224	0.041	1.036
	Linolenic Acid	0.258	0.045	1.016
	Cyclotrisiloxane	0.375	0.046	1.002
KO-cyp_vs_KO	N-Methyltrifluoroacetamide	0.235	0.002	1.625
	Valine	0.120	0.002	1.591
	d-Glucose	0.292	0.004	1.529
	Urea	0.356	0.014	1.523
	alpha-Methylhydroxylamine	0.316	0.007	1.510
	beta-D-Mannopyranose	0.156	0.008	1.448
	methanamine	0.333	0.010	1.426
	Cyclotrisiloxane	0.313	0.015	1.422
	N-Acetyl glucosamine methoxime	0.312	0.006	1.409
	Tris(trimethylsilyl) phosphate	0.361	0.013	1.391
	D-Arabinose	0.256	0.009	1.380
	2-hydroxypropanoic acid	0.346	0.012	1.347

alpha-Monopalmitin	0.361	0.021	1.321
Hexadecanoic acid	0.321	0.020	1.288
Threonine	0.368	0.014	1.211
Phosphonic acid	0.333	0.031	1.205
Putrescine	0.359	0.032	1.180

Notes: FC, fold change; *P*-value, *P*-value < 0.05 from two-tailed Student's T-test; VIP, variable importance in projection value.
