# Supplementary figures and images for: Metabolomics’ Change Under β-Cypermethrin Stress and Detoxification Role of CYP5011A1 in Tetrahymena thermophila
Source: Metabolites. 2025 Feb 20;15(3):143. doi: 10.3390/metabo15030143 (PMC11944115; doi:10.3390/metabo15030143)

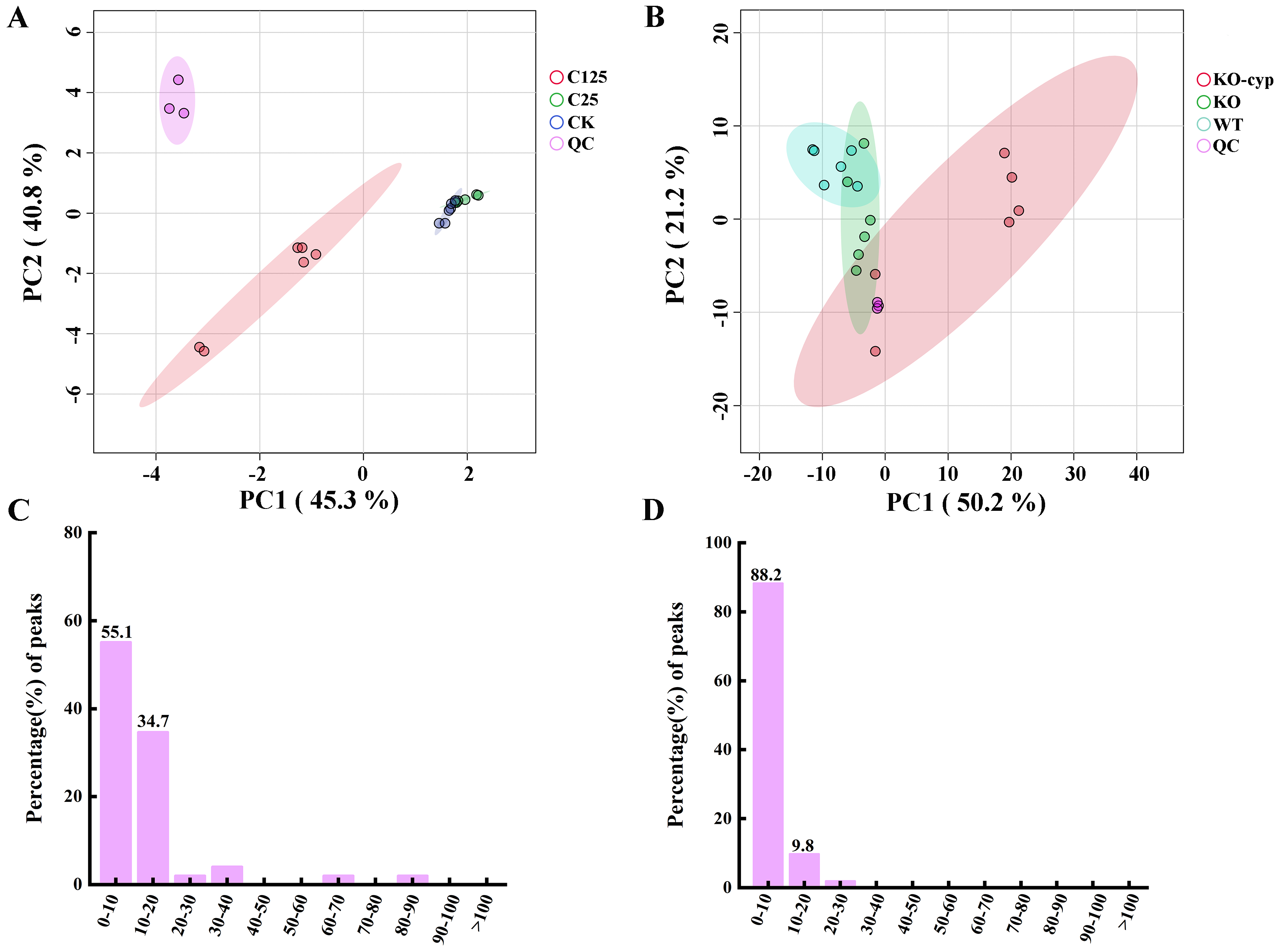

Supplement: Supplementary file 1 [file metabolites-15-00143-s001.zip › Figure Suppl/Figure_S1.tif]

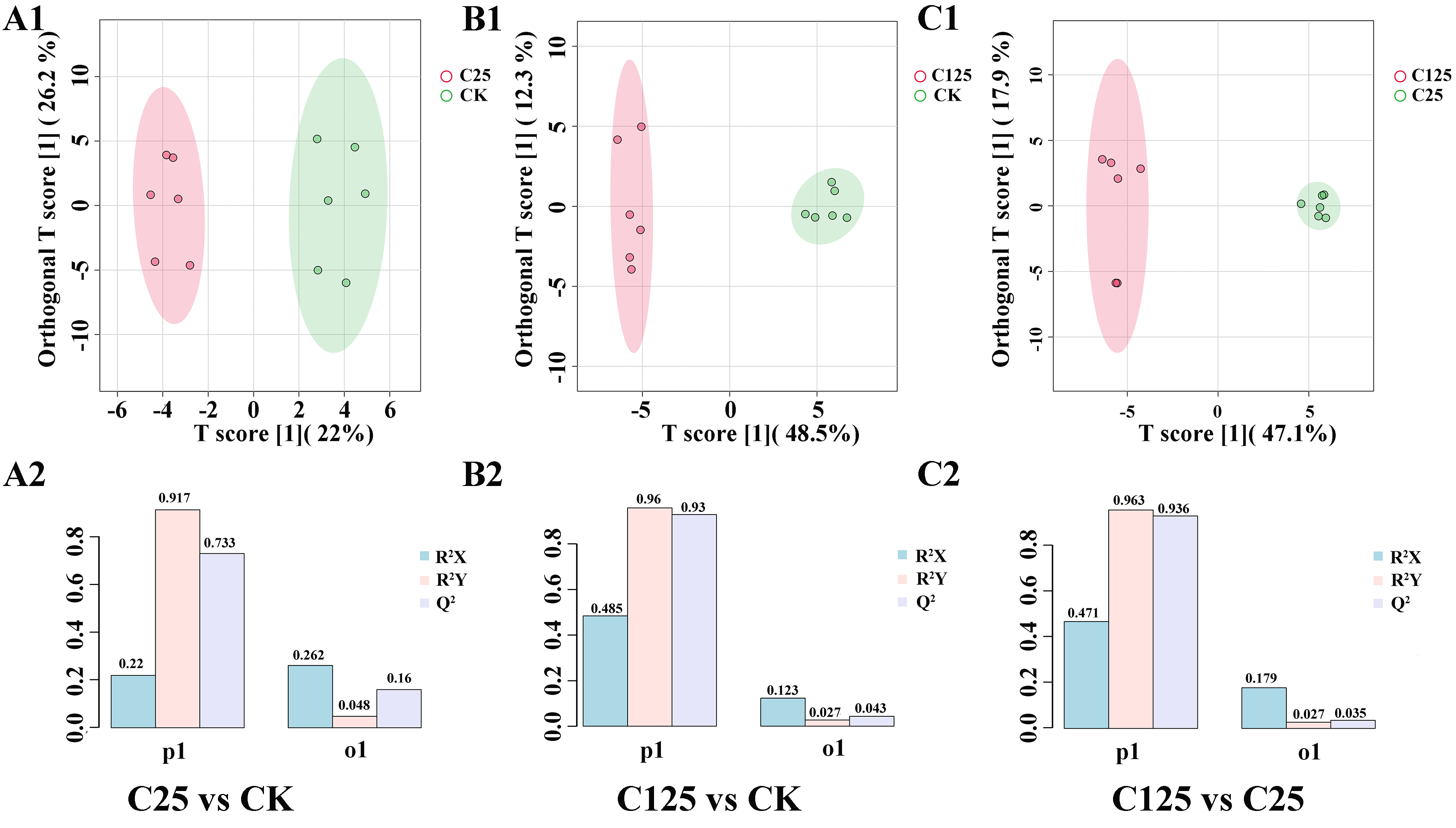

Supplement: Supplementary file 1 [file metabolites-15-00143-s001.zip › Figure Suppl/Figure_S2.tif]

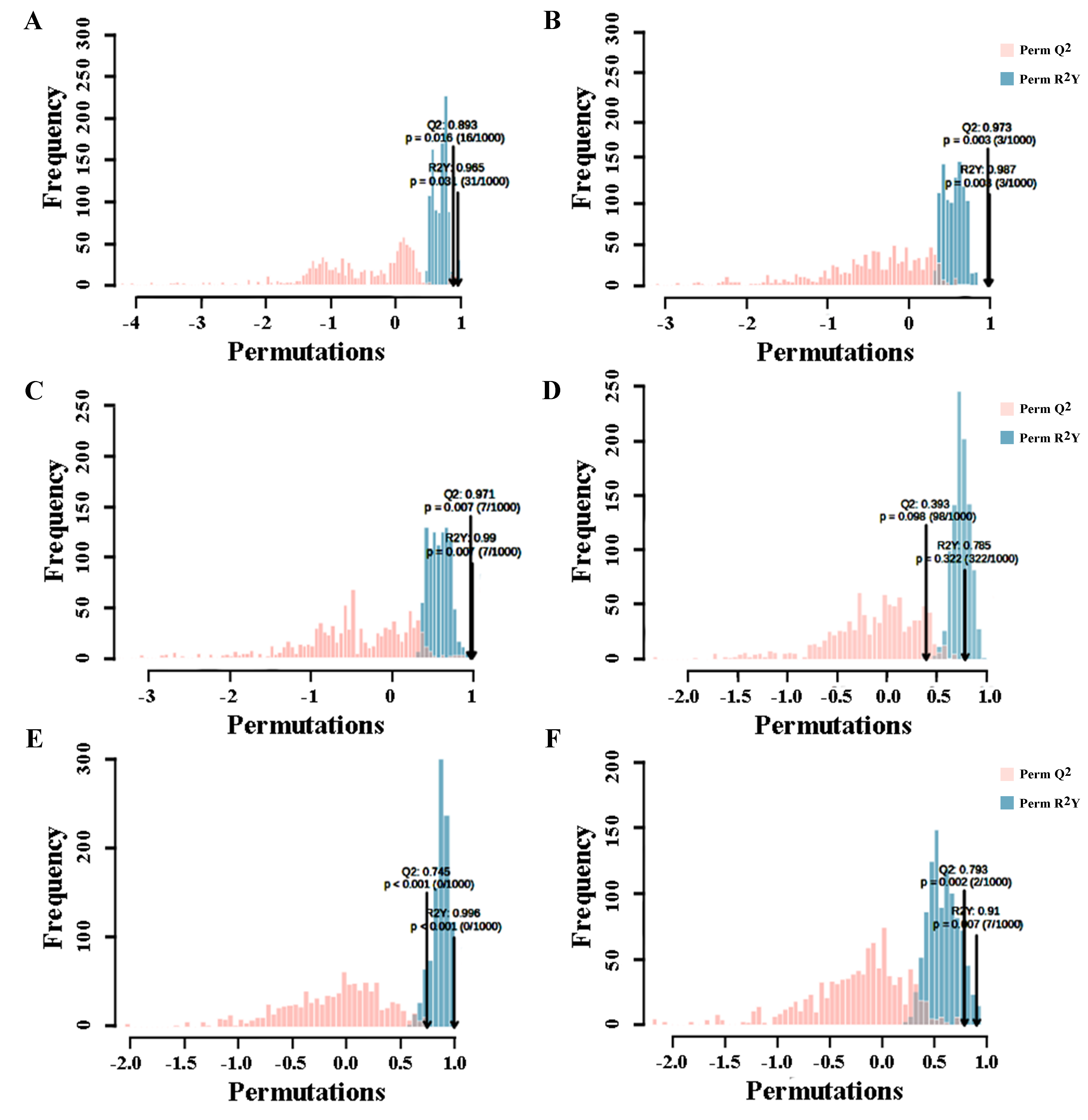

Supplement: Supplementary file 1 [file metabolites-15-00143-s001.zip › Figure Suppl/Figure_S3.tif]

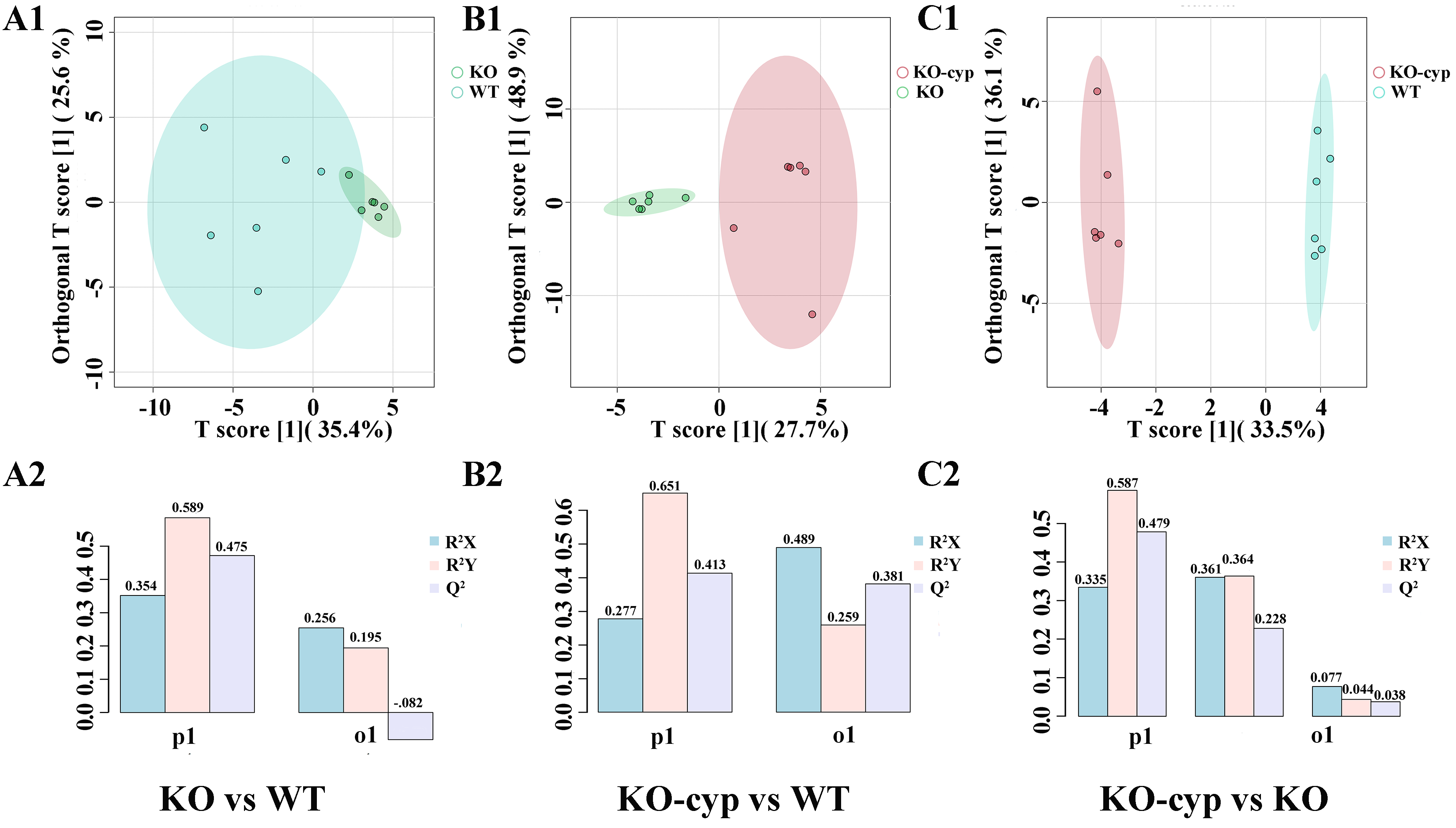

Supplement: Supplementary file 1 [file metabolites-15-00143-s001.zip › Figure Suppl/Figure_S4.tif]

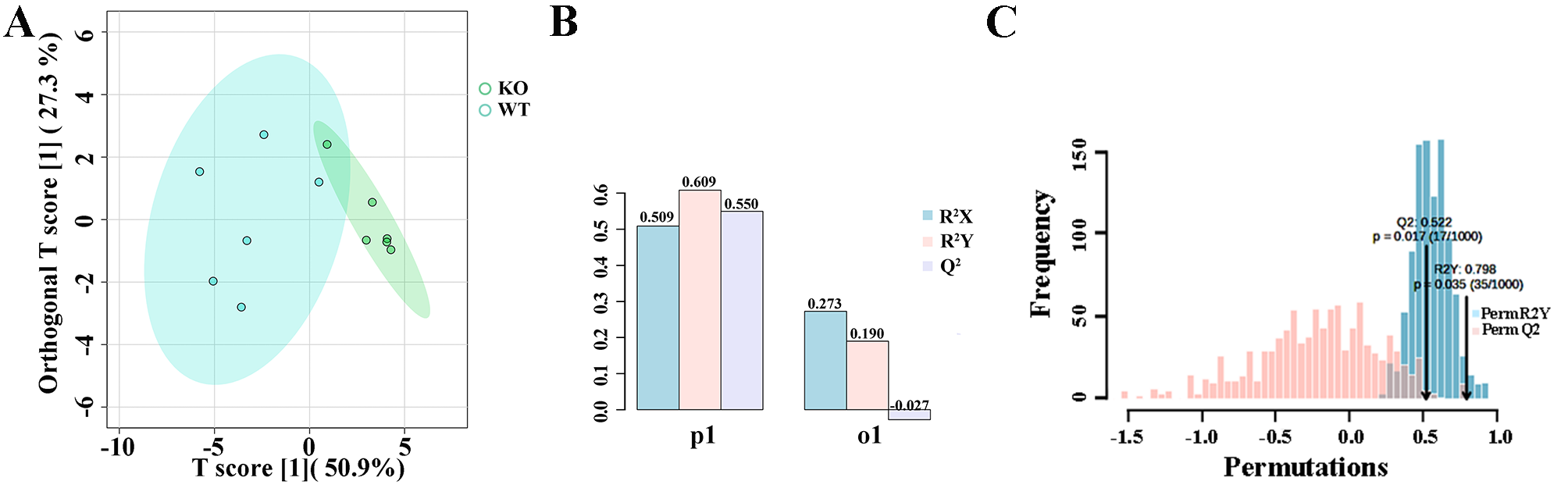

Supplement: Supplementary file 1 [file metabolites-15-00143-s001.zip › Figure Suppl/Figure_S5.tif]

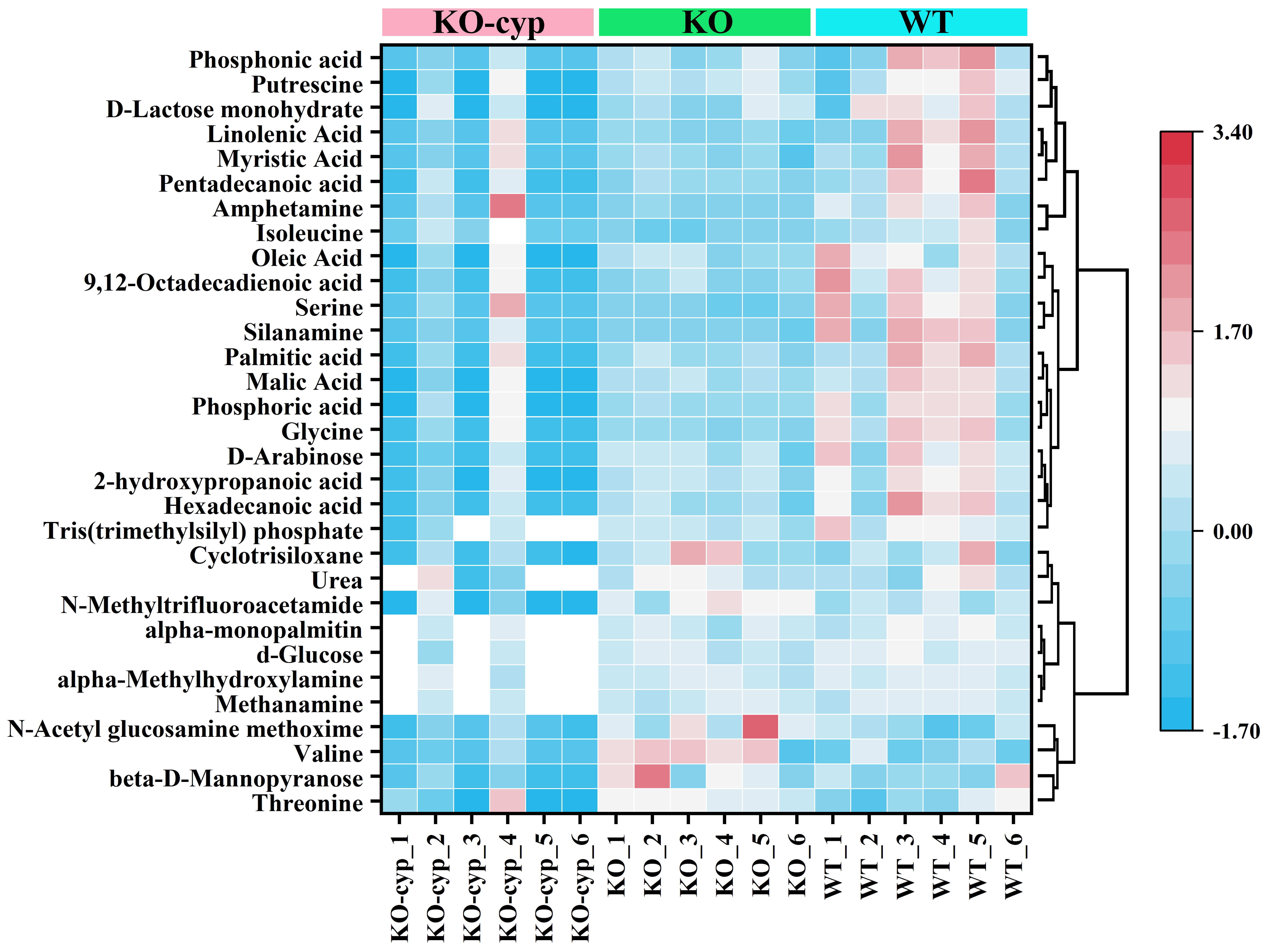

Supplement: Supplementary file 1 [file metabolites-15-00143-s001.zip › Figure Suppl/Figure_S6.tif]

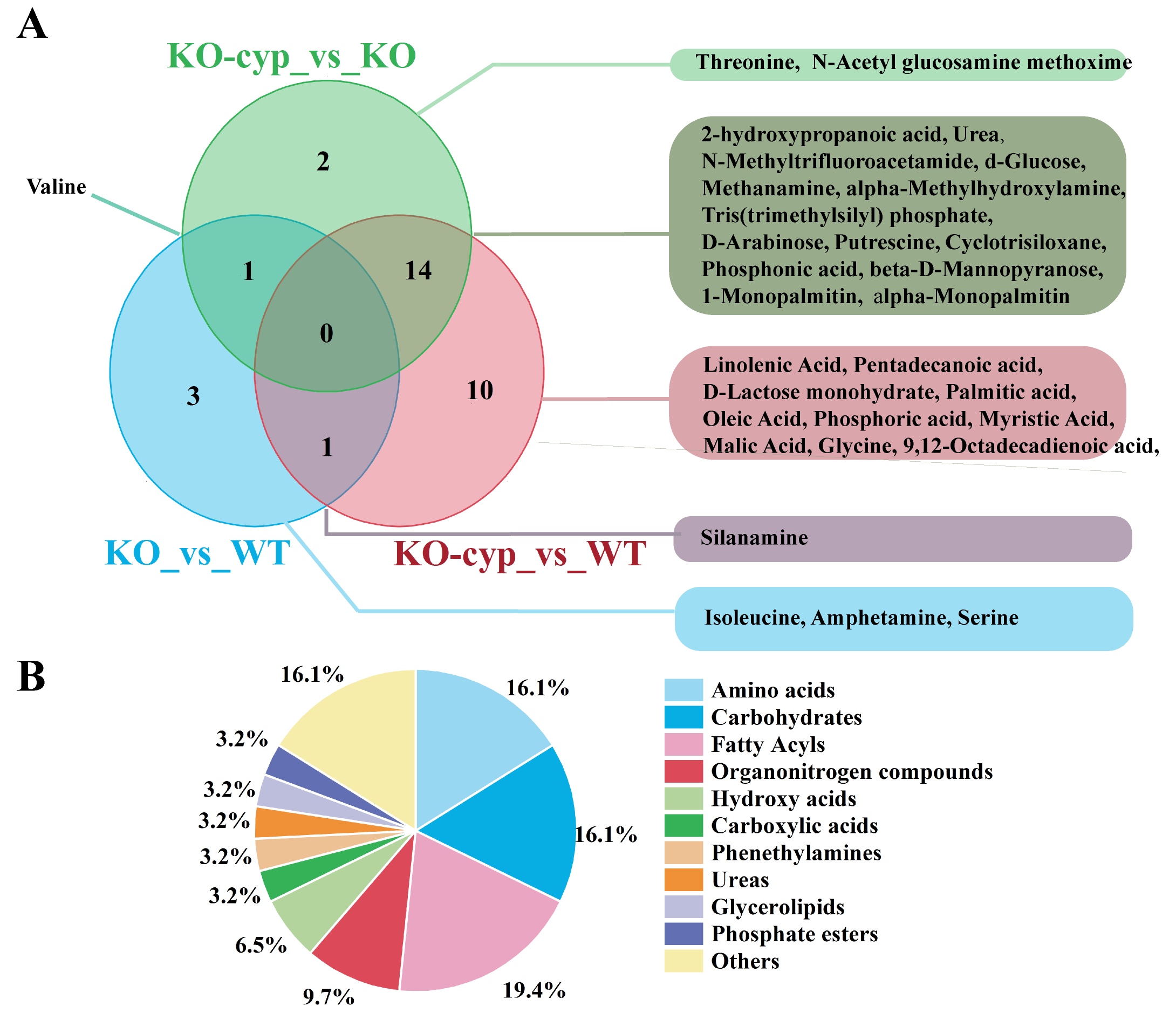

Supplement: Supplementary file 1 [file metabolites-15-00143-s001.zip › Figure Suppl/Figure_S7.tif]

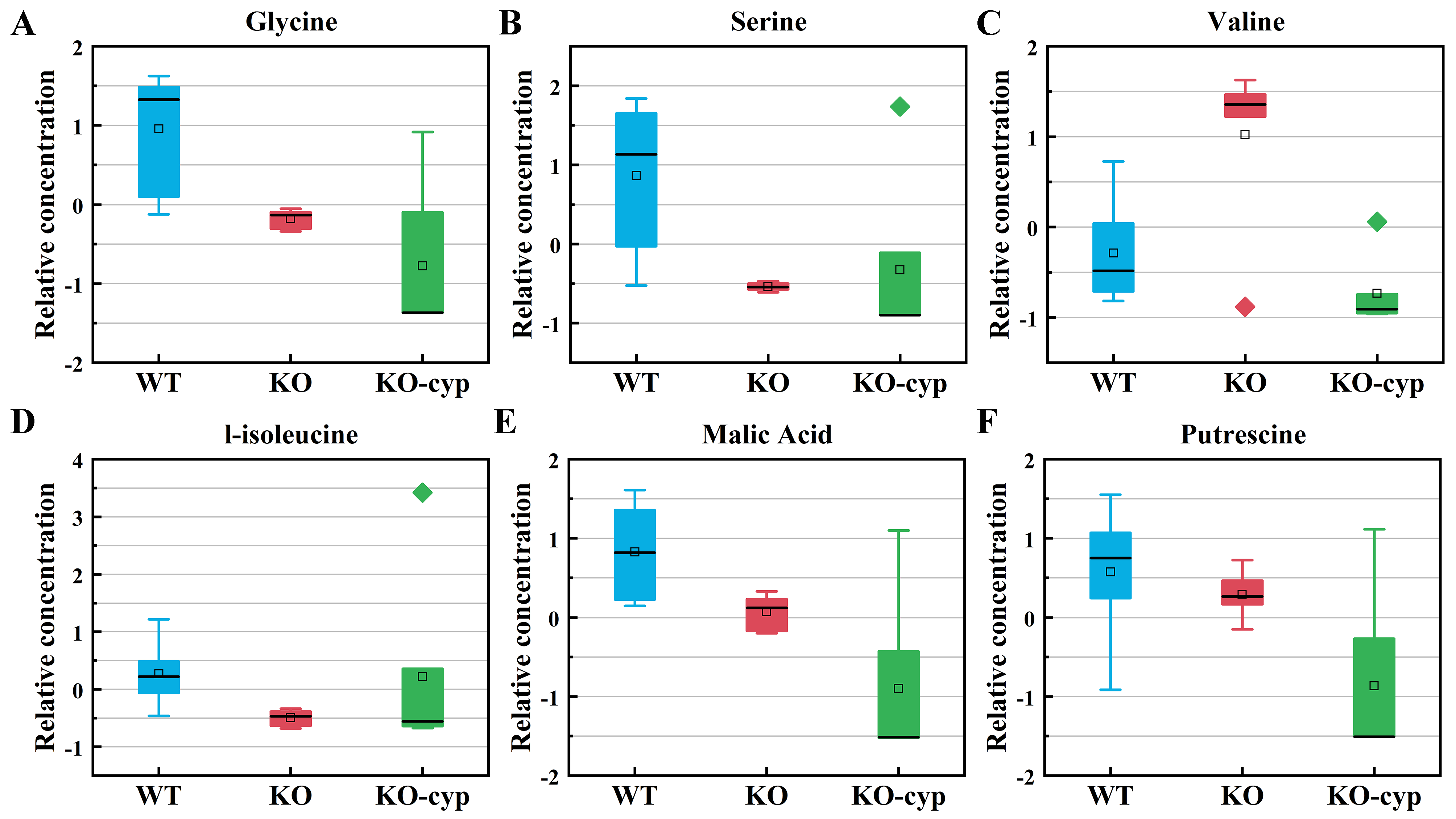

Supplement: Supplementary file 1 [file metabolites-15-00143-s001.zip › Figure Suppl/Figure_S8.tif]
